# Precursor Design Principles for Area‐Selective Atomic Layer Deposition of Alumina: Quantifying the Role of Lewis Acidity, Steric Repulsion, and Dimerization in Surface Adsorption

**DOI:** 10.1002/jcc.70493

**Published:** 2026-09-03

**Authors:** Patrick Maue, Douglas L. Miller, Fabian Pieck, Ralf Tonner‐Zech

**Affiliations:** ^1^ Fakultät für Chemie Wilhelm‐Ostwald‐Institut für Physikalische und Theoretische Chemie, Universität Leipzig Leipzig Germany

**Keywords:** ALD, aluminum, DFT, energy decomposition analysis, precursors

## Abstract

In atomic layer deposition (ALD), precursor adsorption plays a critical role in determining growth characteristics. This is particularly important in area‐selective ALD (AS‐ALD), where adsorption on the non‐growth surface (NGS) leads to selectivity loss. In this context, both the ability of a precursor to access reactive surface groups on the NGS and the strength of its binding are key parameters governing the selectivity in experiment. Adsorption on the NGS is strongly influenced by the precursor's substituents, which control Lewis acidity, steric demand, and propensity for dimerization. We applied energy decomposition analysis for extended systems (pEDA) to a set of commonly used aluminum precursors to quantitatively assess how these factors affect adsorption energetics on SiO_2_ as the NGS, with trimethoxypropylsilane (TMPS) as a small‐molecule inhibitor (SMI). Using fluoride ion affinity (FIA) as a metric for Lewis acidity, we find that this largely governs the trends in precursor bonding to both the surface and the SMIs. Notable exceptions are precursors bearing branched alkyl substituents for which steric repulsion dominates over Lewis acidity. Lewis acidity furthermore determines whether adsorption between the SMI layer remains energetically favorable. In this regime, steric effects—Pauli repulsion and deformation of the SMI layer—substantially weaken the adsorption. Dimerization generally reduces adsorption energies. This weakening effect is less pronounced, however, when adsorption takes place within the inhibitor layer. Based on these insights on bonding energetics, we recommend targeted modifications of existing aluminum precursors and propose systematic strategies for precursor design aimed at minimizing interactions with the NGS.

## Graphical abstract

Key influences on precursor adsorption in AS–ALD.

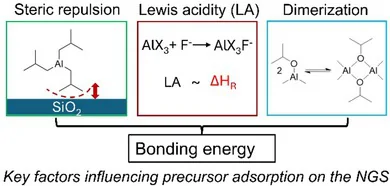

## 1 Introduction

Atomic layer deposition (ALD) is a technique for the controlled deposition of thin films by the sequential application of precursor molecules to a surface where they react and form the desired material [1, 2]. If the deposition takes place selectively on a growth surface (GS) versus an adjacent non‐growth surface (NGS), the process is called area‐selective ALD (AS‐ALD) [3, 4]. Deposition processes—such as the growth of a dielectric material on a metal substrate using a dielectric as the NGS, as considered in this study—are particularly relevant for microelectronics manufacturing [3]. In the currently most often used approach for AS‐ALD, the NGS is protected by applying small molecule inhibitors (SMIs) [5]. A major challenge, especially with SMIs, is that the number of ALD cycles the process can be performed selectively before deposition occurs on the NGS (selectivity window) is limited [4, 5]. To improve AS‐ALD processes, the precursor is a key ingredient as it influences the adsorption behavior [6], growth rates [7] and the achieved selectivity [8, 9, 10]. On a molecular level, the precursor effect on the selectivity is rooted in the chemical structure of the precursor and its ability to penetrate the inhibitor layer, as discussed previously [6, 11, 12]. However, not only chemical reactions but every adsorption of precursors—even physisorption on top of the inhibitor layer [13], can lead to the loss of selectivity. An exemplary experimental observation that underscores the impact of precursor adsorption on selectivity is that chlorinated precursors—which bind stronger to the OH groups of the substrate—exhibit significantly lower selectivity compared to alkylated precursors, unless the purging time is extended [8]. This shows that reversible adsorption, likely occurring on the surface of the SMI layer, plays a crucial role. Due to the higher Lewis acidity of chlorinated precursors, their increased adsorption negatively affects selectivity, unless the adsorbed species are effectively removed.

**FIGURE 1:**
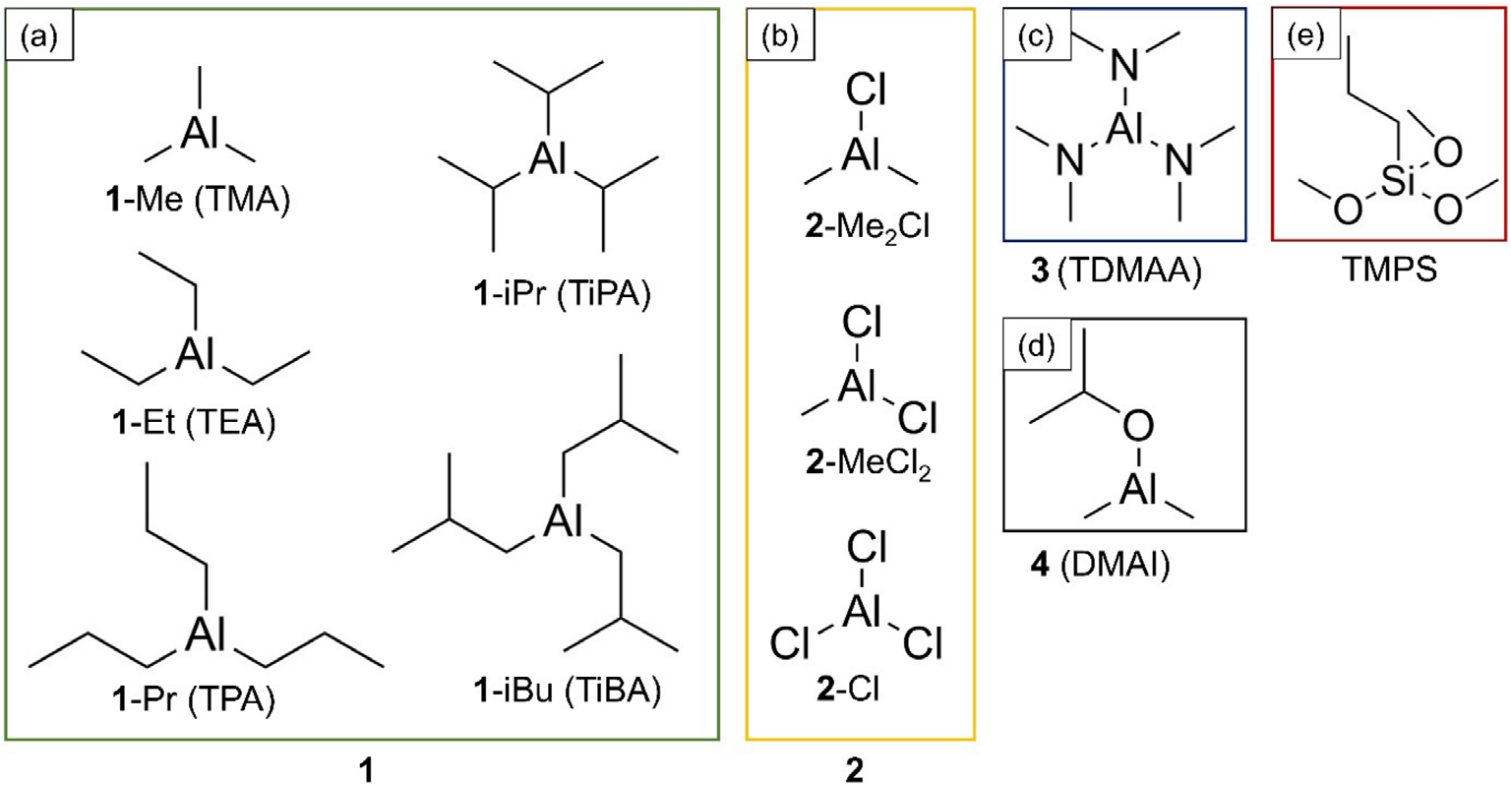
Al‐precursors tested in this study grouped in substance classes **1–4**. (a) Alkyl substituted precursors of class 1. (b) Chlorinated precursors of class **2**. (c) TDMAA, class **3**. (d) DMAI, class **4**. (e) The SMI trimethoxypropylsilane (TMPS). Abbreviations used in the ALD literature are shown in brackets where available. Figure reproduced from Maue et al. [12], 2026, licensed under CC BY 4.0.

In previous projects we investigated a set of aluminum precursors for their tendency to dimerize and to interact with hydroxyl groups on SiO_2_ substrate inhibited with trimethoxypropylsilane (TMPS) as SMI [12, 14]. The molecules are shown in Figure 1 below. These studies gave first indications that dimerization, precursor size, and Lewis acidity play a role for the adsorption behavior, albeit no quantification of their relative importance could be given. We thus set out in this study to systematically and quantitatively analyze the role of Lewis acidity, substitution pattern, steric repulsion and degree of dimerization on precursor adsorption. Furthermore, we investigate whether the bonding energy of a precursor can be predicted from its Lewis acidity and assess when dispersion and steric effects, not adequately captured by Lewis acidity alone, must also be taken into account.

We thus applied the energy decomposition analysis for extended systems (pEDA) to quantitatively investigate the bonding between different precursors and SiO_2_. We compare the results to fluoride ion affinities (FIAs), a common tool to evaluate Lewis acidity. In addition, pEDA allows us to analyze how substitution effects and precursor dimerization affect precursor adsorption. Finally, we consider the full spectrum of factors (Lewis acidity, steric repulsion, and dimerization) and establish design principles for precursors with tailored properties for AS‐ALD.

## 2 Computational Details

### 2.1 Computations for Extended Systems

#### 2.1.1 Structure Optimizations Including the Surface

Periodic slab calculations were performed with VASP 5.4.4 [15, 16, 17, 18, 19], the PBE functional [20] with DFT‐D3(BJ) [21, 22] dispersion correction and a plane wave cutoff of 450 eV using standard PAWs [23]. The Brillouin zone was sampled with a Γ‐centered (2 × 2 × 1) Monkhorst‐Pack [24, 25] mesh. The plane wave cutoff and k‐grid were determined in a convergence test to an accuracy of 1 kJ mol^−1^. The surface model was the 3 × 3 supercell of a crystalline *α*‐quartz slab consisting of three O‐Si‐layers where the bottom layer of silicon was H‐terminated and kept frozen during the optimization. The top oxygen layer was saturated by hydrogen resulting in a fully hydroxylated silica surface. The theoretically optimized cell parameters are *a* = *b* = 14.803 Å, *c* = 52.000 Å. This corresponds to a vacuum above the slab of 45 Å. Minima were optimized with a conjugate gradient algorithm with a convergence criterium for the energy difference of the SCF cycle of 10^−5^ eV. The convergence criterium for the forces within the geometry optimization was set to 10^−2^ eV Å^−1^. The slab was derived from an optimized SiO_2_ bulk cell containing 3 silicon atoms and 6 oxygen atoms and cut along the (001) plane using VESTA 3.5.5 [26]. The bulk structure for optimization was obtained from the Crystallography Open Database [27, 28]. The parameters of the original bulk cell were *a* = *b* = 4.892 Å and *c* = 5.389 Å. The bulk was optimized with an energy cutoff of 500 eV and Γ(5 × 5 × 5) k‐point mesh. The parameters of the optimized bulk cell in the *α*‐quartz phase were *a* = *b* = 4.934 Å and *c* = 5.436 Å. The computational parameters have been derived in previous work on this system [12].

#### 2.1.2 Energy Decomposition Analysis Including the Surface

An energy decomposition analysis for extended systems (pEDA) [29, 30] was performed with AMS‐BAND 2023.104 [31, 32]. As functional, PBE [20] with a DFT‐D3(BJ) [21, 22] dispersion correction was used with the basis set TZ2P [33, 34]. Parameters of the numerical quality were of the level “good” with an SCF‐convergence criterium of $10^{-7}\cdot \sqrt{\left(\text{number of atoms}\right)}\;E_{h}$. Only the Г‐point was considered in all calculations as this was sufficient for an accuracy of 1 kJ·mol^−1^ (see Supporting Information: Section 1, Table 1). Scalar relativistic effects were included using the zeroth‐order regular approximation ZORA [35]. A frozen core approximation of the quality “small” was applied freezing all 1 s orbitals except for H.

The used pEDA is an extension of the EDA, developed by Morokuma [36] as well as Ziegler and Rauk [37, 38], to periodic systems. Here, the bonding energy $E_{\text{bond}}$ between two fragments A and B is separated into the interaction energy $∆E_{\mathrm{int}}$ of these fragments and the preparation energy $∆E_{\text{prep}}$:

(1) $$E_{\text{bond}}=∆E_{\text{prep}}+∆E_{\mathrm{int}}.$$

$E_{\text{bond}}$ is the difference between the energy of the final structure $E_{\mathrm{AB}}$ and the energies of the fragments in their relaxed geometry $E_{A}^{\mathrm{rel}}$ and $E_{B}^{\mathrm{rel}}$:

(2) $$E_{\text{bond}}=E_{\mathrm{AB}}-E_{A}^{\mathrm{rel}}-E_{B}^{\mathrm{rel}}.$$

$∆E_{\text{prep}}$ is the difference between the energies of the fragments in the geometry of the final structure $E_{A}$ and $E_{B}$ and the energies of the fragments in their relaxed geometry $E_{A}^{\mathrm{rel}}$ and $E_{B}^{\mathrm{rel}}$ (Figure 1):

(3) $$∆E_{\text{prep}}=E_{A}+E_{B}-E_{A}^{\mathrm{rel}}-E_{B}^{\mathrm{rel}}.$$

Consequently, the preparation energy represents the change in energy necessary for the deformation of the fragments from their relaxed geometry to the final geometry. The interaction energy is furthermore separated into a dispersion contribution $∆E_{\mathrm{int}}\left(\text{disp}\right)$ and an electronic contribution $∆E_{\mathrm{int}}\left(\text{elec}\right)$:

(4) $$∆E_{\mathrm{int}}=∆E_{\mathrm{int}}\left(\text{disp}\right)+∆E_{\mathrm{int}}\left(\text{elec}\right).$$

The electronic term of the interaction energy is finally decomposed into three different terms, which determine the chemical nature of the bond:

(5) $$∆E_{\mathrm{int}}\left(\text{elec}\right)=∆E_{\text{Pauli}}+∆E_{\text{elstat}}+∆E_{\mathrm{orb}}.$$

The electrostatic term $∆E_{\text{elstat}}$ comprises the quasi‐classical electrostatic interactions between the fragment charge distributions, the Pauli term $∆E_{\text{Pauli}}$ describes the repulsive effect due to normalization and antisymmetrisation of the resulting product wavefunction and the attractive orbital term $∆E_{\mathrm{orb}}$ takes charge transfer and polarization effects into account [30, 39, 40]. More detailed information about the EDA scheme presented here is found elsewhere [41, 42].

### 2.2 Molecular Calculations

#### 2.2.1 Fluoride Ion Affinities

The fluoride ion affinity (FIA) is a common measure for the Lewis acidity. It is defined as the negative reaction enthalpy of the binding between a fluoride ion and a Lewis acid (reaction see Equation 6): [43, 44]

(6) $$A+F^{-}\to {\mathrm{AF}}^{-}.$$

The advantage of using FIAs is that they are a simple, comparable and physically motivated concept of Lewis acidity that virtually eliminates steric effects by using F^−^ as a hard base. The FIA values were calculated for our set of precursors (see Figure 1). The choice of methodology outlined below follows a study on FIA by Sigmund et al. [43] Structures of the Al‐precursors were preoptimized with GFN2‐xTB [45] (using xtb version 6.7.0). Conformers were then sampled with CREST [46, 47] 3.0.0 to determine the lowest energy conformer. The structure of the lowest energy conformer was reoptimized with PBEh‐3c [48] using a def2‐QZVPPD [49] basis set in ORCA 5.0.4 [50, 51]. The densest Becke‐integration grid [52] “defgrid3” was used and SCF cycles were converged to an energy change of 10^−8^ E_h_ in all DFT calculations for the FIA. The geometry was converged to the “verytightopt” threshold (2 × 10^−7^ E_h_ energy change, 8 × 10^−6^ E_h_ bohr^−1^ RMS gradient, 3 × 10^−5^ E_h_ bohr^−1^ the maximum element of the gradient, 10^−4^ bohr RMS step size and 2 × 10^−4^ bohr maximum displacement). The optimized minima were confirmed via frequency [53] calculations. An imaginary mode with −11 cm^−1^ in case of **2**‐MeCl_2_ that remained despite these convergence criteria, was tolerated. With the optimized structures, accurate single point energies were calculated with RI‐DSD‐BLYP [54] with a DFT‐D3(BJ) [21, 22] dispersion correction and a def2‐QZVPPD [49] basis set. The same grid and convergence criteria as for the geometry optimizations were used. An auxiliary basis for coulomb integrals RI‐J [55] and a COSX [56] numerical integration for HF exchange, together referred to as “RIJCOSX” were used. Furthermore, EDA analysis was done on the precursor‐fluoride bond, described in Section 3 in the Supporting Information.

#### 2.2.2 EDA on Fluoride Ion Affinity

EDA analysis of the precursor–fluoride bond was carried out using the Morokuma–Ziegler [36, 37, 38] scheme. Geometry optimizations were performed in AMS 2023.101 [31, 44] with ADF, starting from the structures used in the FIA calculations. Because the double‐hybrid functional DSD‐BLYP is incompatible with EDA, the PBE0 [57] functional was employed instead, as it showed the smallest deviation from DSD‐BLYP in a benchmark of electronic FIA values. All calculations used a DFT‐D3(BJ) [21, 22] dispersion correction and a TZ2P [33, 34] basis set, with numerical quality set to “very good.” Geometries were converged to 10^−3^ E_h_ Å^−1^ (maximum gradient), 6.66 × 10^−4^ E_h_ Å^−1^ (RMS gradient), 0.01 Å (maximum step), 6.66 × 10^−3^ E_h_ Å^−1^ (RMS step), 5 × 10^−5^ E_h_ (energy change), and 5 × 10^−4^ E_h_ (stress). No frozen‐core approximation was used. EDA calculations were performed at the same level of theory, with the complex partitioned into F^−^ and the Al‐precursor fragment.

#### 2.2.3 Partial Charges

Hirshfeld charges [58] were calculated using ORCA 5.0.3 [50, 51]. Single‐point calculations were performed on the geometries optimized for the determination of the FIA. The PBEh‐3c method [48] was employed in conjunction with the def2‐QZVPPD basis set [49], using the densest Becke integration grid (“defgrid3”) [52]. SCF calculations were converged to an energy change threshold of 10^−8^ E_h_.

## 3 Results and Discussion

First, the trends in Lewis acidity of the different precursors are presented in Section 3.1. Section 3.2 discusses the EDA results as a function of substitution pattern. Also, the correlation between FIA and the individual EDA contributions is analyzed while the effects of dimerization and the SMIs blocking layer are discussed. Finally, in Section 3.3, the impact of substitution on precursor adsorption energetics is placed in a broader context to propose design principles for optimized precursors.

### 3.1 Trends in Lewis Acidity

The FIA is taken as a common measure to quantify Lewis acidity in order to check for its influence on the adsorption behavior of precursors. It is defined as the negative reaction enthalpy of the binding between a fluoride ion and a Lewis acid (Equation 6) [43, 44]. The values are listed in Table 1 for each precursor (see Figure 1) in monomeric form where higher FIA values represent a stronger Lewis acidity.

**TABLE 1:** FIAs of the precursors shown in Figure 1.

Precursor	FIA^a^	Relative FIA^b^
**2**‐Cl	495	144
**2**‐MeCl_2_	446	95
**2**‐Me_2_Cl	403	52
**1**‐iPr	388	37
**1**‐iBu	382	31
**1**‐Pr	371	20
**1**‐Et	368	17
**4**	362	11
**1**‐Me	358	7
**3**	351	0

^a^ FIA in kJ mol^−1^ computed with DSD‐BLYP‐D3/def2‐QZVPP//PBEh‐3c/def2‐QZVPP.

^b^ FIA relative to **3** in kJ mol^−1^.

The result shows that the chlorinated precursors **2** have the highest FIA, which increases with the number of Cl atoms from 403 to 495 kJ·mol^−1^. The alkyl substituted precursors with branched groups, **1**‐iPr and **1**‐iBu, come next with 382 and 388 kJ·mol^−1^ followed by those with the linear alkyl groups ranging from 358 to 371 kJ·mol^−1^ (FIA decreases with decreasing alkyl chain length). **4** has a slightly higher (4 kJ·mol^−1^) FIA than **1**‐Me, while **3** has with 351 kJ·mol^−1^ the lowest FIA of all precursors. It is 144 kJ·mol^−1^ lower than that of **2**‐Cl, with the highest FIA, which underlines the large spread in Lewis acidity among the Al precursors.

It is chemically intuitive that the Lewis acidity increases with the number of chlorine atoms due to the electron withdrawing effect resulting in the chlorinated precursors **2** being stronger Lewis acids than the alkyl substituted precursors **1**. This is further reflected in the progressive shortening of the bond length and the corresponding increase in orbital overlap with an increasing number of chlorine substituents, as summarized in Table S2.

However, it is counterintuitive at first glance that precursors with branched groups have higher FIAs than those with linear alkyl chains, or that the FIA increases with the length of the alkyl group. One might expect, as often discussed in organic chemistry literature, that alkyl groups show a +I‐effect, which is stronger for branched than linear alkyl chains and that also increases with the chain length. Such a +I‐effect would lead to a lower FIA. To understand the present trend, it needs to be considered that aluminum is more electropositive than carbon. Consequently, alkyl groups bonded to aluminum show a −I‐effect. Therefore, the trends are as expected for aluminum‐carbon bonds. Note the comparably low FIA of **3** and **4** which have oxygen and nitrogen as heteroatoms. While those are more electronegative than carbon, they have nonbonding electron pairs which can donate electrons into the empty p‐orbital of the aluminum thus stabilizing the precursor against coordination of a fluoride ion. To gain deeper understanding of the observed FIA trends, we performed an EDA of the bond between each precursor and the fluoride ion which is shown in the following Table 2.

**TABLE 2:** EDA of the precursor‐fluorine bond of the adducts of the precursors with F^−^.^a^

	**2**‐Cl‐F	**2**‐MeCl_2_‐F	**2**‐Me_2_Cl‐F	**1**‐Me‐F	**1**‐Et‐F	**1**‐Pr‐F	**1**‐iPr‐F	**1**‐iBu‐F	**3**‐F	**4**‐F
ΔE_int_	−620	−565	−511	−456	−467	−472	−481	−478	−496	−468
ΔE_int_ (disp)	−3	−3	−3	−3	−4	−3	−5	−5	−4	−3
ΔE_int_ (elec)	−617	−562	−508	−453	−463	−469	−476	−473	−492	−465
ΔE_Pauli_	447	443	421	388	399	402	408	405	452	415
ΔE_elstat_	−748 (70%)	−698 (69%)	−641 (69%)	−576 (68%)	−581 (67%)	−582 (67%)	−588 (67%)	−580 (66%)	−622 (66%)	−596 (68%)
ΔE_orb_	−316 (30%)	−307 (31%)	−288 (31%)	−265 (32%)	−281 (33%)	−289 (33%)	−296 (33%)	−298 (34%)	−322 (34%)	−284 (32%)
ΔE_prep_	93	86	80	74	73	78	68	72	117	80
E_bond_	−527	−479	−431	−382	−394	−394	−413	−406	−379	−388

^a^ Energies in kJ mol^−1^.

The $E_{\text{bond}}$ from the EDA reproduces the trend in FIAs. It should be noted that when we discuss EDA contributions, the absolute values are meant. Otherwise, for the discussion of the overall energies $∆E_{\mathrm{int}}$ and $E_{\text{bond}}$, which have a negative sign, we will make it clear whether they are “stabilized” or “destabilized,” and so forth, to avoid ambiguity.

The EDA rationalizes the higher FIA of precursors bearing branched alkyl groups (**1**‐iPr, **1**‐iBu) compared to their linear analogues, particularly the isomeric pair **1**‐iPr and **1**‐Pr. Although stronger steric shielding might be expected for branched substituents, $∆E_{\text{Pauli}}$ is only slightly increased for **1**‐iPr (6 kJ mol^−1^ relative to **1**‐Pr). This is overcompensated by increased $∆E_{\text{elstat}}$ (by 6 kJ mol^−1^) and $∆E_{\mathrm{orb}}$ (by 7 kJ mol^−1^) contributions, resulting in a stronger $∆E_{\mathrm{int}}$ for the **1**‐iPr–fluoride complex. In addition, **1**‐Pr requires a higher $∆E_{\text{prep}}$ (by 10 kJ mol^−1^). Thus, enhanced orbital overlap, stronger electrostatic interactions, and lower deformation energy account for the higher FIA of **1**‐iPr. The stronger attractive interactions likely arise from inductive effects of the branched alkyl groups: due to the low electronegativity of aluminum, secondary carbon substituents withdraw more electron density from the metal center, increasing its affinity toward fluoride.

A further trend is the increase in FIA with increasing alkyl chain length for precursors bearing linear substituents. From **1**‐Me to **1**‐Pr, the $∆E_{\text{prep}}$ increases by 4 kJ mol^−1^ and $∆E_{\text{Pauli}}$ by 14 kJ mol^−1^. However, these destabilizing effects are overcompensated by increasingly favorable $∆E_{\text{elstat}}$ and $∆E_{\mathrm{orb}}$ contributions, resulting in a stronger $E_{\text{bond}}$ and, consequently, a higher FIA. This behavior can be rationalized analogously to the trend observed for branched alkyl substituents: longer alkyl chains exert a stronger inductive effect, withdrawing more electron density from the aluminum center and thereby enhancing its attractive interaction with fluoride.

Finally, precursors **3** and **4** exhibit lower FIAs than the other compounds, with **3** showing the lowest value in the series (only **1**‐Me is slightly lower than **4**). At first glance, this may appear counterintuitive, as the electron‐withdrawing character of oxygen and nitrogen substituents would be expected to decrease the electron density at the aluminum center. However, these heteroatoms can also exert a +M effect, donating electron density into the vacant p orbital of aluminum in the isolated precursor. The EDA reveals that both **3** and **4** display more favorable $∆E_{\text{elstat}}$ contributions than the alkyl‐substituted precursors of group **1**, and **3** also shows a higher $∆E_{\mathrm{orb}}$ contribution. Nevertheless, these stabilizing effects are outweighed by significantly higher $∆E_{\text{Pauli}}$ and $∆E_{\text{prep}}$, resulting in overall weaker bonding and thus lower FIAs. This behavior supports the hypothesis that the increased electron density at aluminum arising from the +M donation of the heteroatom lone pairs must be displaced upon fluoride coordination and therefore leads to enhanced Pauli repulsion. As the discussed examples illustrate, trends in Lewis acidity are not always intuitive. It is therefore essential to rely on quantitative measures such as FIA or EDA rather than solely on qualitative or intuitive assessments in chemical discussions.

### 3.2 Bonding Analysis of Precursor Adsorption

**FIGURE 2:**
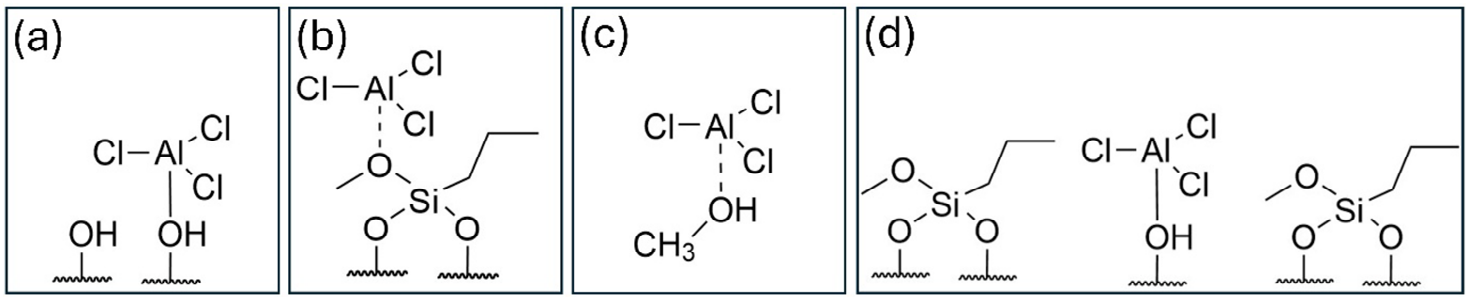
Investigated adsorption structures of precursors illustrated at the example of **2**‐Cl. (a) Adsorption to the free SiO_2_ surface. (b) Adsorption to a methoxy group of the SMI (TMPS) on the SiO_2_ surface. (c) Adsorption to methanol as comparison to (b). (d) Adsorption on the partially TMPS‐covered surface in between the inhibitor molecules.

Understanding how substituents influence the bonding between precursor and NGS is important for precursor design. To investigate the substituent effects as well as the effect of surrounding inhibitors on the bonding, EDA analyses were performed for different adsorption structures relevant for the AS‐ALD process (Figure 2). The EDA shows by the individual terms how the nature of the bond changes with the different substituents. This helps to identify whether Lewis acidity, steric repulsion or other effects are responsible for the trend in bonding energy. The EDA values are then compared with the FIAs to investigate the correlation between these values. The analysis aims to determine under which conditions effects such as dispersion or steric repulsion, which are not sufficiently captured by the FIA, exert a significant influence on the bonding energies in addition to Lewis acidity.

First, we will analyze how the substitution of the precursor in the monomeric form influences $E_{\text{bond}}$ comparing adsorption on free SiO_2_ surface (Figure 2a) and TMPS‐passivated surface (Figure 2b) and discuss the correlation with the FIA values as measure for the Lewis acidity. In a second step we will consider dimerization and its influence on the chemical bond of the precursor in these two bonding situations. Finally, we have a closer look on how the presence of surrounding SMIs affects the bond between precursor and surface (Figure 2d). In addition to these structures a comparison of structure (b) with the adsorption to methanol in structure (c) is presented in Section 4 in the Supporting Information. With this comparison, we can test the influence of the surface on the observed trends in the bonding of the precursors to methoxy groups.

#### 3.2.1 Number of Chlorine Substituents

**FIGURE 3:**
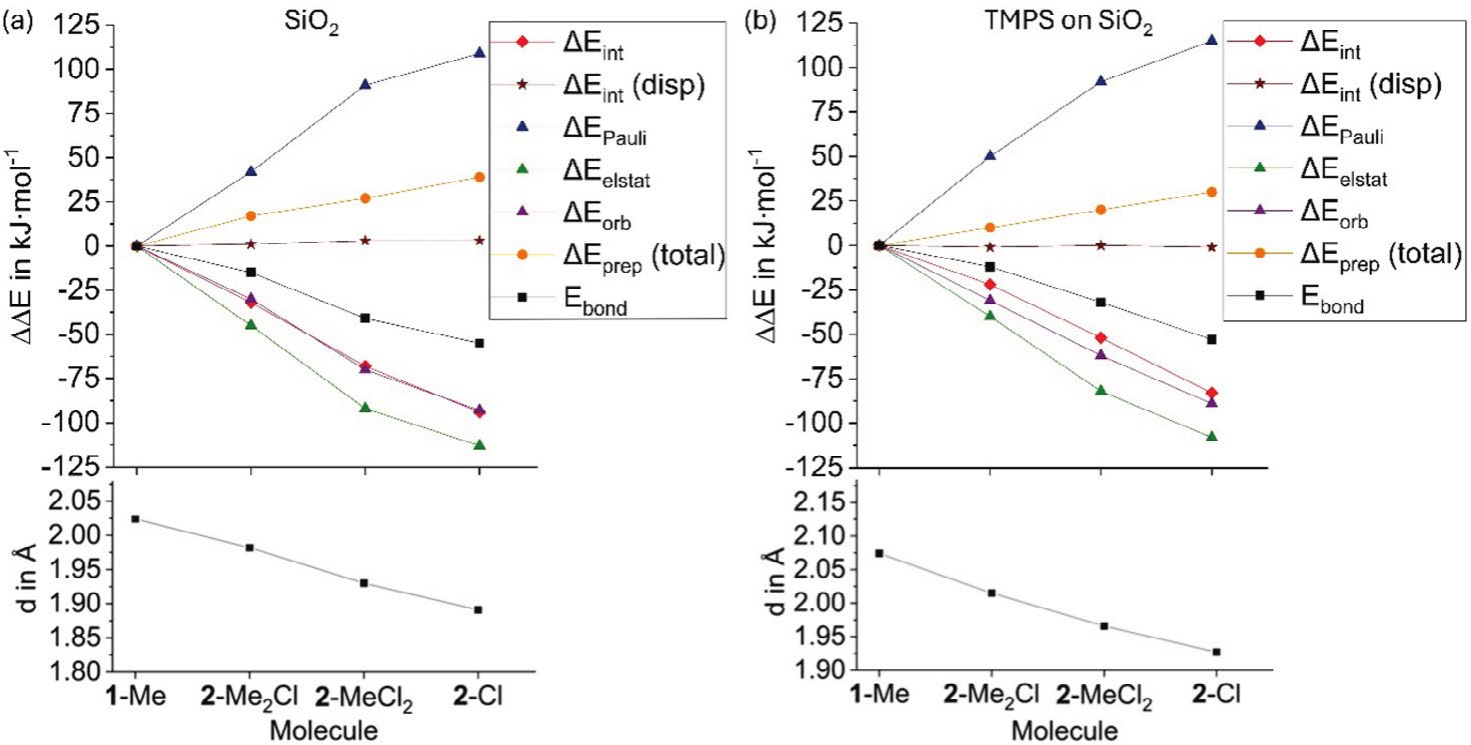
pEDA terms relative to **1**‐Me for the chlorinated precursors of group **2** on (a) SiO_2_ (Figure 2a) and (b) TMPS on SiO_2_ (Figure 2b).

The first chemical parameter tested is the number of chlorine atoms. We address the question of how the bond changes with increasing number of chlorine atoms and to which degree this change is determined by the Lewis acidity. All EDA results of Section 3.2 are listed in Tables S3–S11 in Sections 5 and 6 in the Supporting Information. Here, only the relative trends will be discussed. Figure 3 shows the terms relative to **1**‐Me for the adsorption to SiO_2_ (Figure 3a) and to TMPS on SiO_2_ (Figure 3b).

For the adsorption to SiO_2_ (see Figure 3a, Table S3) it can be observed that $E_{\text{bond}}$ stabilizes with increasing number of Cl ligands by 55 kJ mol^−1^ from **1**‐Me to **2**‐Cl. $∆E_{\mathrm{int}}\left(\text{disp}\right)$ changes insignificantly (≤ 3 kJ mol^−1^). The $∆E_{\text{Pauli}}$ contribution increases by 109 kJ mol^−1^ to **2**‐Cl, while the $∆E_{\text{elstat}}$ and $∆E_{\mathrm{orb}}$ contributions increase by 113 and 123 kJ mol^−1^, respectively. As a sum of attractive and repulsive terms, $∆E_{\mathrm{int}}$ gets strengthened by 94 kJ mol^−1^. $∆E_{\text{prep}}$ of the precursors increases by 16 and that of the surface by 23 kJ mol^−1^. The bond length becomes shorter with increasing number of Cl by 0.133 Å from **1**‐Me to **2**‐Cl. All EDA contributions, except dispersion, continuously increase with the number of Cl substituents.

The trends for the adsorption to the methoxy group of TMPS (see Figure 3b, Table S9) are essentially the same as those for the adsorption to SiO_2_ as Figure 3b shows. That means that the precursor binds stronger to the surface and to TMPS, the more Cl atoms it contains. This is the case because the increase of the attractive contributions $∆E_{\text{elstat}}$ and $∆E_{\mathrm{orb}}$ with increasing number of Cl dominates over the increase of the contributions that weaken the bond, which are $∆E_{\text{prep}}$ and $∆E_{\text{Pauli}}$. Furthermore, Cl and methyl lead to a comparable $∆E_{\mathrm{int}}\left(\text{disp}\right)$ and therefore dispersion does not significantly contribute to the trend.

The result show that the more Cl ligands a precursor contains, the more energy is needed to deform precursor and surface into the bonding geometry. Nevertheless, this is overcompensated by the attractive interaction due to electrostatics and orbital interactions between precursor and the oxygen of the surface or inhibitor. This confirms the expectation that Lewis acidity, which is reflected in the electrostatic and orbital terms, determines the trend in $E_{\text{bond}}$ for the chlorinated precursors of class **2**. A plausible chemical explanation is that the chlorine substituent withdraws electron density from the aluminum center, thereby increasing its partial positive charge. This enhances the electrostatic attraction to the fluoride ion, shortens the bond distance, and promotes stronger orbital interaction. This interpretation is consistent with a moderate trend seen in the partial charges reported in Table S4.

#### 3.2.2 Length and Branching of Alkyl Substituents

**FIGURE 4:**
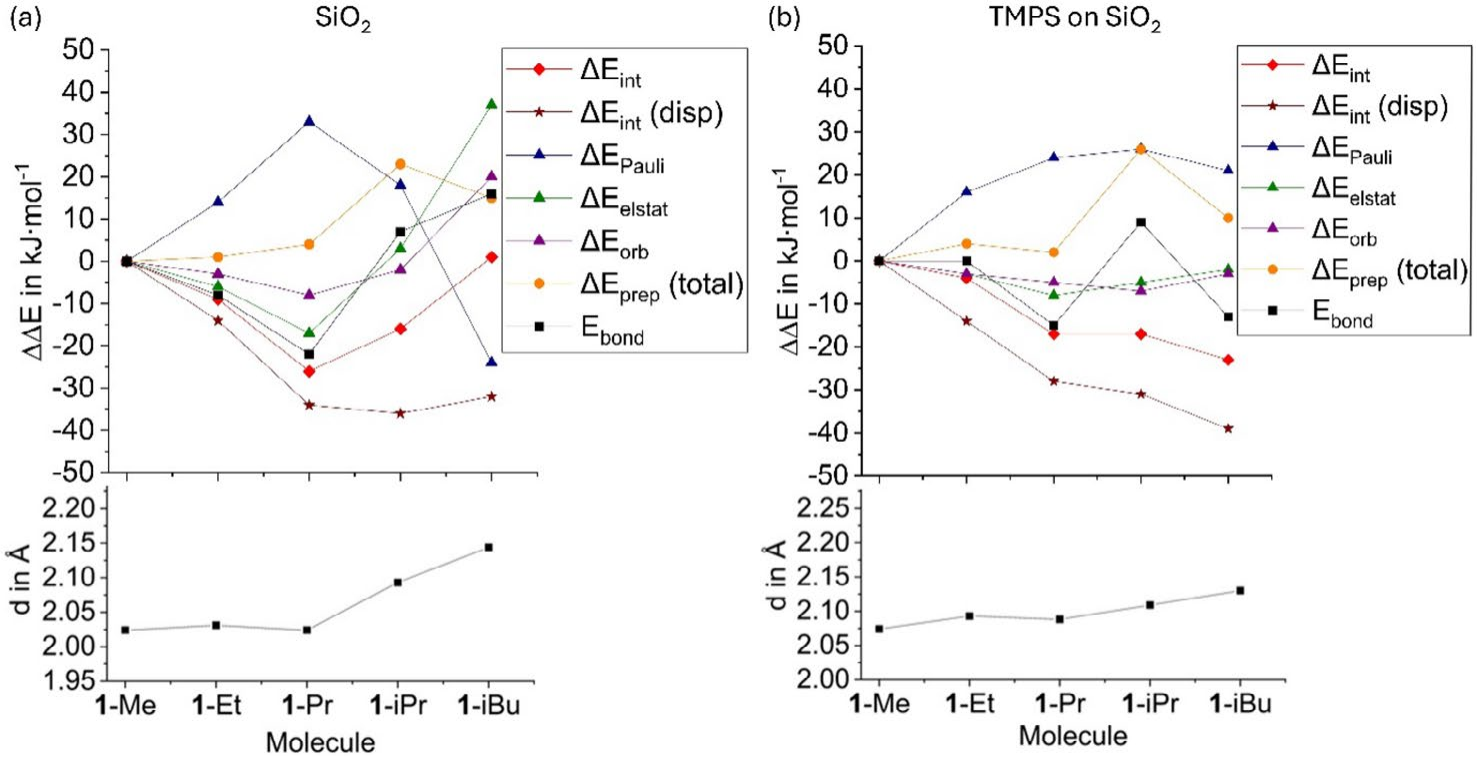
pEDA terms relative to **1**‐Me for the alkyl substituted precursors **1** on (a) SiO_2_ (Figure 2a) and (b) TMPS on SiO_2_ (Figure 2b).

Another parameter that determines the Lewis acidity and especially the steric repulsion of the precursors is the chain length and branching of alkyl substituent. The order of Lewis acidity as determined by FIA (Table 1) is: **1**‐iPr > **1**‐iBu > **1**‐Pr > **1**‐Et > **1**‐Me. The order of steric bulkiness is assumed to be **1**‐iPr/**1**‐iBu > **1**‐Pr > **1**‐Et > **1**‐Me. Although the iso‐butyl group possesses the larger overall size, the iso‐propyl group contains a secondary carbon atom directly bound to the aluminum center. In the context of S_N_2‐reactivity, substituents featuring secondary α‐carbon atoms are generally considered sterically more hindering [59], suggesting that **1**‐iPr may provide stronger steric shielding of the aluminum center toward coordination. On a planar surface, however, the influence of the overall substituent size may become more significant. Consequently, no unambiguous assignment regarding the relative steric repulsion is made here. Table S5 shows the EDA of the alkyl substituted precursors **1** on SiO_2_. The corresponding table for the adsorption to TMPS can be found in Table S10. Figure 4 shows the relative terms for the adsorption to SiO_2_ and to the methoxy group of TMPS. Because of the steric bulk of **1**‐iPr and **1**‐iBu, the influence of different conformers on the adsorption energies was evaluated by calculating the adsorption energies of the 10 lowest‐energy conformers identified by CREST (see Section 2.2.1). The corresponding results, together with the selection of the most favorable adsorption structures, are presented in Tables S7 and S8. The figure below displays the EDA trends obtained using the selected conformers. For comparison, Figure S7 presents the EDA results based on the original conformers of **1**‐iPr and **1**‐iBu. The trends are similar in both cases.

First, we have a look at the effect of chain length for the linear alkyl groups on SiO_2_ (Figure 4a, Table S5). Here, $E_{\text{bond}}$ stabilizes by 22 kJ mol^−1^ from **1**‐Me to **1**‐Pr. The attractive $∆E_{\mathrm{int}}\left(\text{disp}\right)$ contribution increases by 34 kJ mol^−1^. Over the same series, $∆E_{\text{Pauli}}$ contribution increases by 33 kJ mol^−1^, while the attractive contributions $∆E_{\text{elstat}}$ and $∆E_{\mathrm{orb}}$ increase by 17 and 8 kJ mol^−1^, respectively. The overall interaction energy $∆E_{\mathrm{int}}$ stabilizes by 26 kJ mol^−1^. In contrast, $∆E_{\text{prep}}$ varies by ≤ 4 kJ mol^−1^ and does not influence the trend. The results mean that, for linear groups, bond strength increases with chain length, reflecting progressively enhanced attractive interactions, predominantly arising from dispersion contributions. Although this trend is consistent with the observed order of Lewis acidity, the enhanced dispersion interactions—which are not fully captured by the FIA descriptor—constitute the primary driving force behind the increase in bonding strength.

Second, the effect of branched groups on SiO_2_ (Figure 4a, Table S5) is investigated. As the effect of branching is most visible for similar precursor size, we want to start with the comparison of **1**‐Pr and **1**‐iPr. Here, $E_{\text{bond}}$ is destabilized by 29 kJ mol^−1^, while no effect of branching on the dispersion interaction is visible. In contrast, $∆E_{\text{Pauli}}$, $∆E_{\mathrm{orb}}$ and $∆E_{\text{elstat}}$ contributions are substantially reduced by 15, 6, and 20 kJ mol^−1^, respectively. This leads to a destabilization of $∆E_{\mathrm{int}}$ by 10 kJ mol^−1^. Consistent with these trends, the bond length increases by 0.069 Å. For **1**‐iBu the observed trends in bond length and energies are continued in comparison to the smaller **1**‐iPr. These data show that branched groups exhibit longer bond distances and reduced interactions with the surface and, consequently, overall weaker bonding compared to their linear counterparts. Consequently, the increase in Lewis acidity is overruled by steric repulsion indicated by longer bonds for these precursors. This already shows that neither Lewis acidity nor steric repulsion alone govern $E_{\text{bond}}$ for the alkyl precursor series. Although branched substituents exhibit higher Lewis acidity, they bind less strongly than expected. This discrepancy can be attributed to steric effects: The bulkier groups hinder close approach to the surface, resulting in elongated bond distances and reduced electrostatic and orbital interactions. Consequently, steric repulsion weakens adsorption and overrides the intrinsic increase in Lewis acidity relative to linear analogues.

Finally, we compare the trends observed on SiO_2_ with those for adsorption at the methoxy group of TMPS (Figure 4b, Table S10) to check the influence of the substrate in comparison to molecular reactivity. For linear groups, again a stabilization of the bonding energy with increasing alkyl chain length is obtained. However, the influence of branching differs from that on SiO_2_. This time, when going from **1**‐Pr to **1**‐iPr no trend reversal nor large changes are present for $∆E_{\text{elstat}}$, $∆E_{\mathrm{orb}}$, and $∆E_{\text{Pauli}}$. This difference can be attributed to reduced steric constraints at the methoxy group, which allow bulky ligands to approach the oxygen atom more closely and thereby diminish the steric penalty associated with branching as reflected in similar bond lengths. This is even more pronounced—in tendency very similar—when the surface is completely neglected using methanol as the model system (Figures 2c and S3–S6). $E_{\text{bond}}$ remains weakened for the adsorption to TMPS. Interestingly, this weakening is less pronounced for **1**‐iBu than for **1**‐iPr. This difference arises primarily from the stronger dispersion interaction for **1**‐iBu, which contributes an additional 9 kJ·mol^−1^, together with its lower $∆E_{\text{prep}}$, by 8 kJ·mol^−1^. Figure S8 compares the adsorption geometries of **1**‐iBu and **1**‐iPr on TMPS, providing a structural rationale for the enhanced dispersion interaction observed for **1**‐iBu.

In summary, for this class of precursors bearing linear alkyl substituents, the trend in $E_{\text{bond}}$ is consistent with their Lewis acidity, although dispersion interactions constitute the dominant contribution.

#### 3.2.3 Precursors 3 and 4 With O and N as Heteroatoms

**FIGURE 5:**
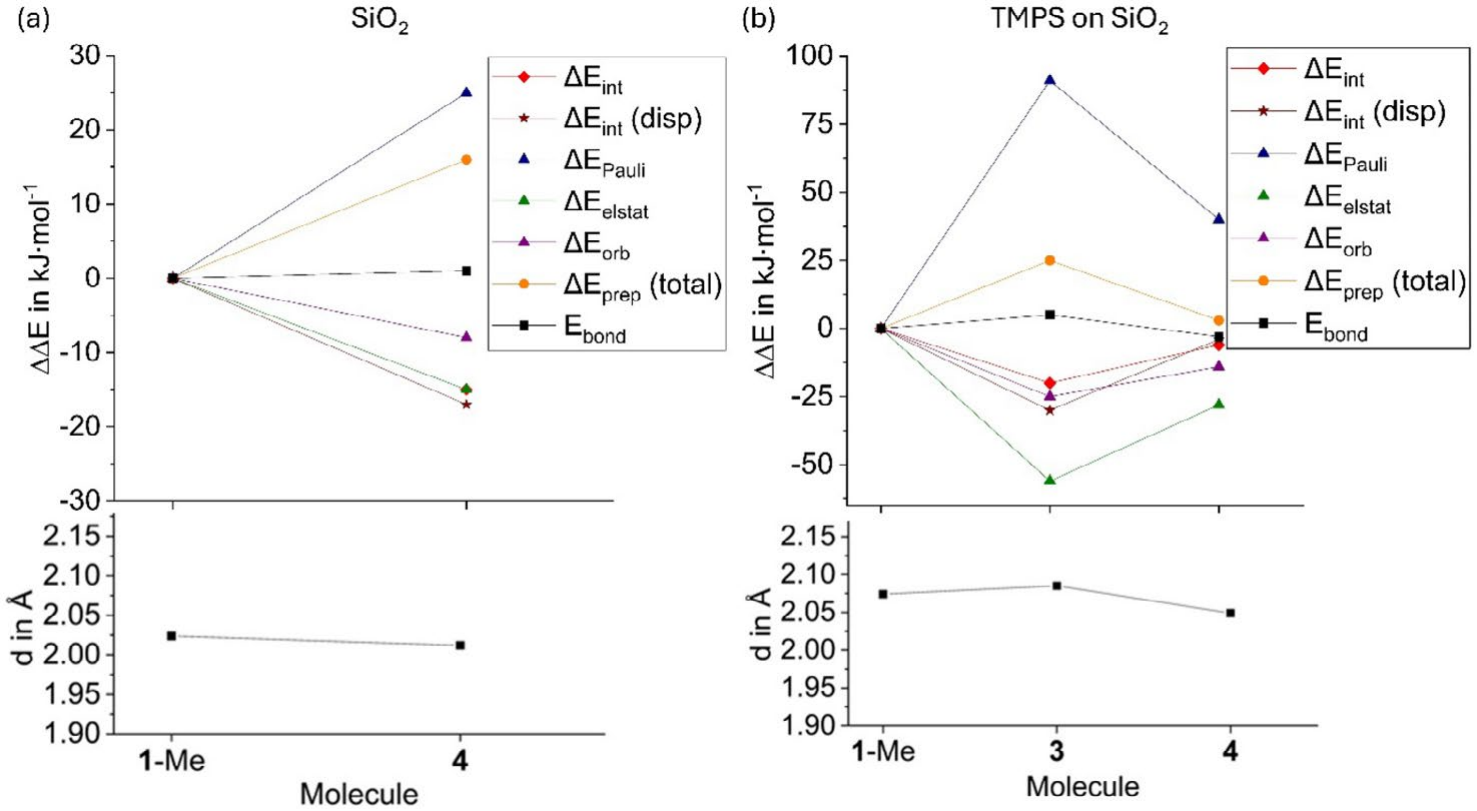
(a) Relative pEDA terms to **1**‐Me. **4** adsorbing to SiO_2_. (b) Relative pEDA terms to **1**‐Me for **3** and **4** adsorbing to TMPS on SiO_2_.

Precursors **3** and **4** are weaker Lewis acids compared to groups **1** and **2**. Again adsorption structures on SiO_2_ and on the methoxy group of TMPS on SiO_2_ were calculated. **3**, however, does not only form a bond between the aluminum and the surface oxygen on SiO_2_, but a proton of the surface is transferred to the nitrogen atom of the dimethylamino‐substituent (see Figure S2). This renders the fragmentation in the EDA step not meaningful. Therefore, this structure is not included in the bonding analysis and only the EDA terms of **4** relative to **1**‐Me are shown in Figure 5a.

To elucidate the effect of oxygen as a heteroatom we begin with the comparison of **1**‐Me and **4** on SiO_2_ (Figure 5a, Table S6). Both precursors exhibit the same $E_{\text{bond}}$, despite notable differences in the individual EDA components ranging from 8 kJ mol^−1^ ($∆E_{\mathrm{orb}}$) to 25 kJ mol^−1^ ($∆E_{\text{Pauli}}$). This means that the nature of the bond changes, but compensation between the individual contributions results in essentially identical bond strengths. This is consistent with the small difference in the FIA values of **1**‐Me and **4**, which amounts to only 4 kJ mol^−1^.

Although precursor **4** exhibits stronger electrostatic and orbital interactions, this enhancement does not translate into increased overall bond strength. Additional factors at the surface, including dispersion interactions and steric repulsion, which are not fully captured by the FIA, largely offset one another.

To assess whether the same conclusion applies to precursor **3** and to adsorption on TMPS, we compare the trends in the EDA terms for the adsorption to the methoxy group (see Figure 5b and Table S11). Precursor **4** exhibits behavior analogous to that observed for adsorption on the SiO_2_ surface: relative to **1**‐Me, the attractive contributions ($∆E_{\mathrm{int}}\left(\text{disp}\right)$, $∆E_{\text{elstat}}$, and $∆E_{\mathrm{orb}}$) as well as the destabilizing contributions ($∆E_{\text{prep}}$ and $∆E_{\text{Pauli}}$) increase. As a result, the overall bonding energy remains comparable, differing by only 2 kJ mol^−1^. For precursor **3**, $E_{\text{bond}}$ is weakened by only 5 kJ mol^−1^ compared to **1**‐Me. Although the relative trends in the individual EDA terms are similar, the absolute changes are more pronounced than in the case of precursor **4**. Overall, these findings confirm that the FIA remains a reliable descriptor for $E_{\text{bond}}$, consistent with the behavior observed on SiO_2_.

In summary, the comparable or slightly higher bonding energies of **3** and **4** are consistent with their Lewis acidities as determined by FIA values. Other factors, such as dispersion interactions and steric repulsion, do not alter this trend despite the substantial size differences between **3**, **4**, and **1**‐Me. This behavior arises from compensation between dispersion attraction and Pauli repulsion, leaving Lewis acidity as the dominant factor determining the trend.

#### 3.2.4 Correlation Between EDA and FIA

**FIGURE 6:**
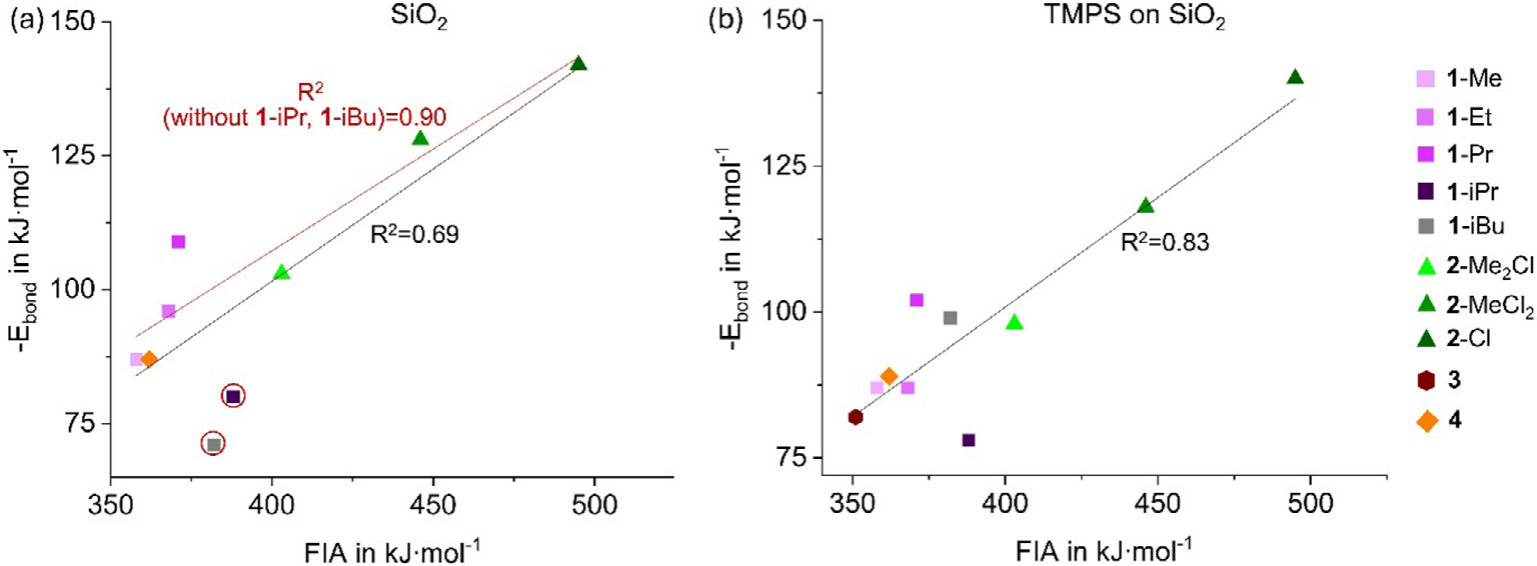
(a) Bonding energy versus FIA for the tested set of precursors adsorbing to SiO_2_ and (b) for adsorption to TMPS on SiO_2_. Linear fit parameters listed in Tables S12 and S13.

After analyzing the EDA terms and the effect of the substituents for each class of precursor, we have a look at how well the result of the EDA correlates with the FIA values taking all precursors and both bonding motifs into account. By this we want to determine how reliable the FIA is as a measure for the adsorption strength of the precursors. Therefore, we primarily test for a correlation with the $E_{\text{bond}}$ term as shown in Figure 6. In addition, to understand which interaction weakens or strengthens the correlation between the FIA and $E_{\text{bond}}$ term the correlation of individual EDA terms with the FIA values is discussed in Section 7 in the Supporting Information.

As a rule of thumb, an *R* ^2^ value of larger than 0.7 indicates the presence of a “strong” correlation [60]. In Figure 6, the coefficient of determination (*R* ^2^) between FIA and $E_{\text{bond}}$ is 0.69 for the adsorption to SiO_2_. If we exclude the largest outliers, which are the branched alkyls **1**‐iPr and **1**‐iBu, a significantly better value of 0.90 is observed. For the adsorption to TMPS (Figure 6b) a *R* ^2^ value of 0.83 is obtained. These results show that especially on the surface, where steric repulsion matters the most, the precursors with branched groups **1**‐iPr and **1**‐iBu lead to deviations from the ideal correlation between the FIA and $E_{\text{bond}}$ values. If we look at the interaction energy $∆E_{\mathrm{int}}$ and leave $∆E_{\text{prep}}$ out which is not so well captured by the FIA we observe a better correlation with *R* ^2^ of 0.90 with and 0.96 without **1**‐iPr and **1**‐iBu (see Figure S9a) on SiO_2_. Of the individual EDA terms ($∆E_{\text{Pauli}}$, $∆E_{\text{elstat}}$ and $∆E_{\mathrm{orb}}$, see Figures S10–S12), $∆E_{\mathrm{orb}}$ correlates best and $∆E_{\text{Pauli}}$ the least with the FIA. This is also expected as the orbital interaction depends less on size or steric effects than $∆E_{\text{Pauli}}$ and $∆E_{\text{elstat}}$ and therefore is captured the most by FIA. This demonstrates that FIA provides a reliable measure of the electronic contributions to bonding between the precursor and surface oxygen, independent of steric effects imposed by the surface.

#### 3.2.5 Impact of Dimerization on Precursor Adsorption

**FIGURE 7:**
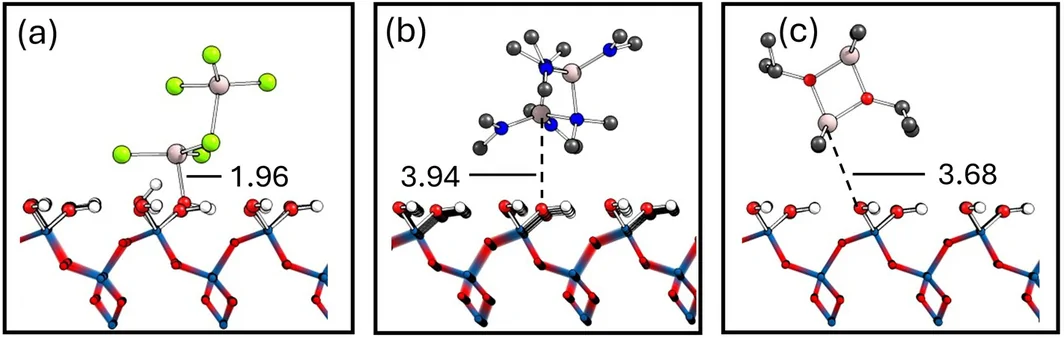
Different adsorption structures of the dimers. (a) (**2**‐Cl)_2_ (b) (**3**)_2_ (c) (**4**)_2_ on SiO_2_. Bond lengths in Å.

Apart from substitution pattern, precursor dimerization (which also depends on the substitution and the ALD conditions) is an important factor that influences the surface chemistry of precursors. As briefly discussed in our previous work, dimers tend to interact less with the surface or inhibitors. We furthermore concluded that compound classes **2**, **3** and **4** are fully or partially present as dimers under common ALD conditions whereas the alkyl substituted precursors **1** are monomeric [14]. It should be mentioned here that dimerization refers to the state of the precursor in gas phase in the ALD reactor in which it reaches the surface. As Figure 7 shows, the chlorinated dimer (**2**‐Cl)_2_ forms a chemical bond to the surface whereas the dimers (**3**)_2_ and (**4**)_2_ are only physisorbed.

**FIGURE 8:**
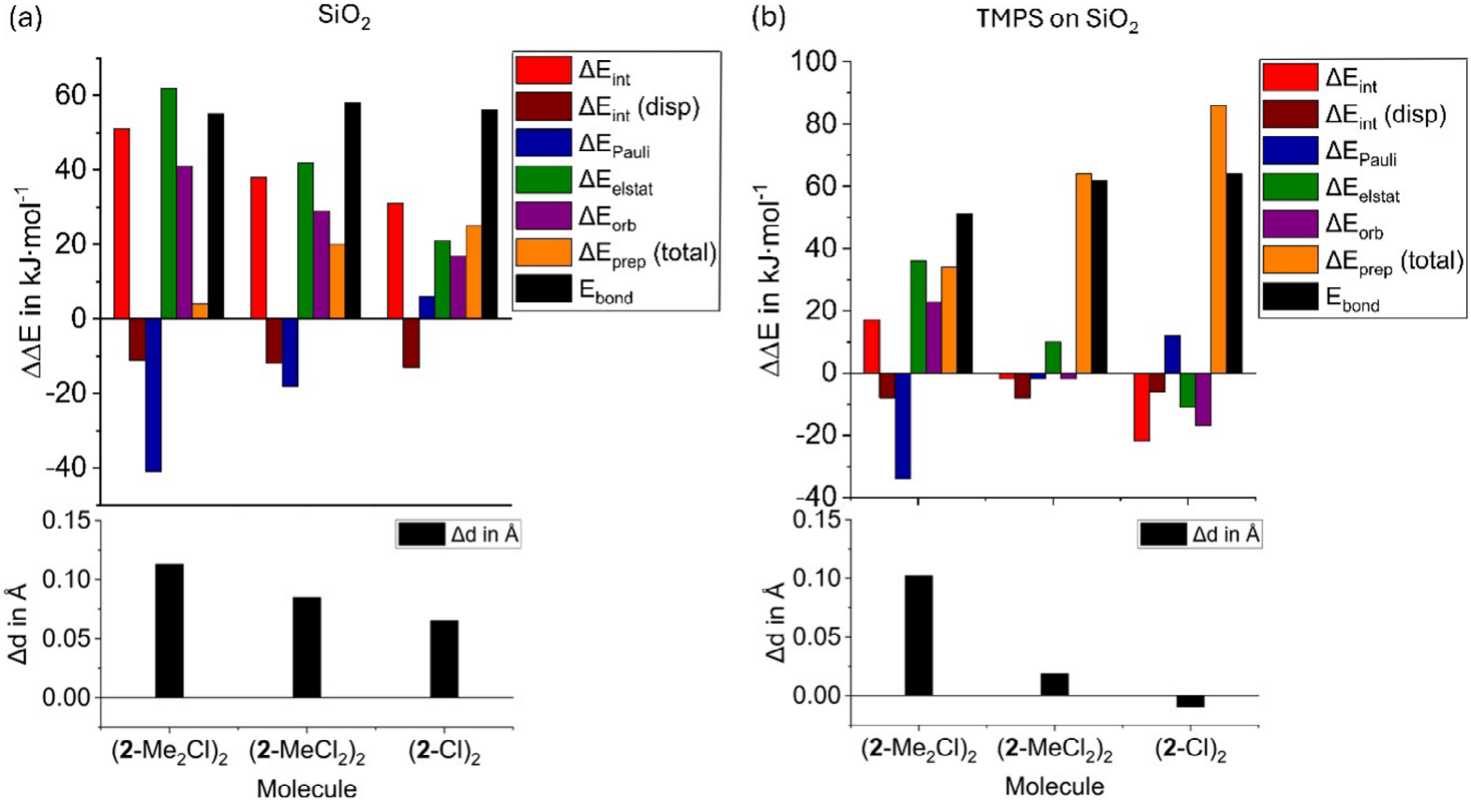
Relative pEDA terms of the dimers of the chlorinated precursors **2** to their corresponding monomers on (a) SiO_2_ (Figure 2a). (b) On TMPS on SiO_2_ (Figure 2b). Δ*d* is the relative bond distance of the dimer compared to the monomer.

As the EDA analysis of a physisorbed molecule does not provide much insight, we focus on the dimers of the chlorinated compound class **2** to address the question of how the bonding energy as well as individual contributions change due to dimerization. Table S14 shows the EDA of monomers and dimers of the chlorinated precursors of group **2** on SiO_2_, and Table S15 shows the EDA for the adsorption to TMPS. Figure 8 visualizes the differences of the EDA terms between dimers and their corresponding monomers for each molecule.

The comparison of monomers and dimers on SiO_2_ (Figure 8a, Table S14) shows that $E_{\text{bond}}$ is weakened by dimerization. For (**2**‐Me_2_Cl)_2_, for example, it is weakened by 55 kJ mol^−1^ compared to the monomer. The $∆E_{\mathrm{int}}\left(\text{disp}\right)$ contribution is higher in case of the dimers, with a 13 kJ mol^−1^ difference for (**2**‐Cl)_2_ versus **2**‐Cl. $∆E_{\text{Pauli}}$ varies, being higher for (**2**‐Cl)_2_ (+8 kJ mol^−1^) and lower for (**2**‐Me_2_Cl)_2_ (−41 kJ mol^−1^). Both, the $∆E_{\text{elstat}}$ and $∆E_{\mathrm{orb}}$ contributions are reduced in case of the dimers, with the smallest differences being 21 kJ mol^−1^ ($∆E_{\text{elstat}}$) and 17 kJ mol^−1^ ($∆E_{\mathrm{orb}}$) for (**2**‐Cl)_2_. The dimers also require a higher $∆E_{\text{prep}}$ for the precursor, while $∆E_{\text{prep}}$ for the surface is higher in case of the monomers (see Table S14). Overall, $∆E_{\text{prep}}$ for the dimers is by 47 kJ mol^−1^ higher (e.g., for (**2**‐Cl)_2_), and bond lengths to the surface are also larger, with 0.113 Å difference for (**2**‐Me_2_Cl)_2_. The increased $∆E_{\text{prep}}$ and weakened $∆E_{\text{elstat}}$, and $∆E_{\mathrm{orb}}$ contributions for the dimers outweigh the enhanced $∆E_{\mathrm{int}}\left(\text{disp}\right)$ and reduced $∆E_{\text{Pauli}}$.

These results show that the dimers adsorb weaker to the surface than their corresponding monomers. The weaker adsorptions are caused by the opening of the dimers necessary to form the bond to the surface as shown in larger $∆E_{\text{prep}}$ values but also by an internally weaker interaction of the opened dimers with the surface as shown by larger bond lengths and weaker $∆E_{\mathrm{int}}$ values. The weakening of the bond is even the more relevant factor as changes to $∆E_{\mathrm{int}}$ are always larger than changes to $∆E_{\text{prep}}$.

When the dimers adsorb to the methoxy group of TMPS (Figure 8b, Table S15), the individual terms behave a bit differently. First, the $∆E_{\text{prep}}$ difference between monomers and dimers is larger, ranging from a difference of 34 kJ mol^−1^ for (**2**‐Me_2_Cl)_2_ to 86 kJ mol^−1^ for (**2**‐Cl)_2_, compared to a maximum of 25 kJ mol^−1^ difference on SiO_2_. This means that more energy is needed to deform the structures, particularly to open the dimer. Second, the $∆E_{\text{elstat}}$ and $∆E_{\mathrm{orb}}$ contributions are less weakened, or even stronger on TMPS compared to SiO_2_. For (**2**‐Cl)_2_, the $∆E_{\text{elstat}}$ contribution is even 11 kJ mol^−1^ and the $∆E_{\mathrm{orb}}$ contribution is 17 kJ mol^−1^ higher compared to the monomer, meaning attractive interactions are stronger for the dimer. However, the enhanced interactions are counterbalanced by the higher $∆E_{\text{prep}}$, resulting in weakened $E_{\text{bond}}$ (51–64 kJ mol^−1^ difference) for the dimers, similar to the destabilization observed on SiO_2_ (55–58 kJ mol^−1^ difference).

In summary, the bond is weakened to a comparable extent upon adsorption on TMPS compared to SiO_2_. To rationalize the distinct behavior of the individual energy contributions, it needs to be considered that the methoxy group enables a more extensive opening of the dimeric structure. This interpretation is supported by the increased Al–Cl bond distances (Table S16). The structural opening permits additional attractive interactions, leading to enhanced electrostatic and orbital contributions. However, these stabilizing effects are offset by the higher $∆E_{\text{prep}}$ required for dimer opening. Consequently, although the intrinsic interaction ($∆E_{\mathrm{int}}$) with the surface may be stronger such as in case of (**2**‐Cl)_2_, the overall bonding energy is reduced to a similar extent as observed for free SiO_2_.

Overall, dimers are weaker adsorbed than the monomers. Opening the dimer requires energy, and in most cases, the opened dimer still shows a weaker Al‐surface interaction than the monomer.

#### 3.2.6 Adsorption Between Inhibitors

The final question is the impact of the surrounding SMI inhibitor layer on the adsorption of the precursor as this is highly relevant for penetration reactions which lead to loss of selectivity. As discussed in our previous study on SMI coverage, this kind of bonding situation is especially relevant when it comes to undesired reactions of the precursor with surface hydroxyl groups [12]. We thus use the previously established model of the TMPS‐covered surface to perform EDA on the adsorption structures of the precursors in between the SMI layer. However, this study is limited to **1**‐Me and the monomeric and dimeric chlorinated precursors **2**, as only these precursors led to stable adsorption structures (see Figures S13, S14, and 2d).

**FIGURE 9:**
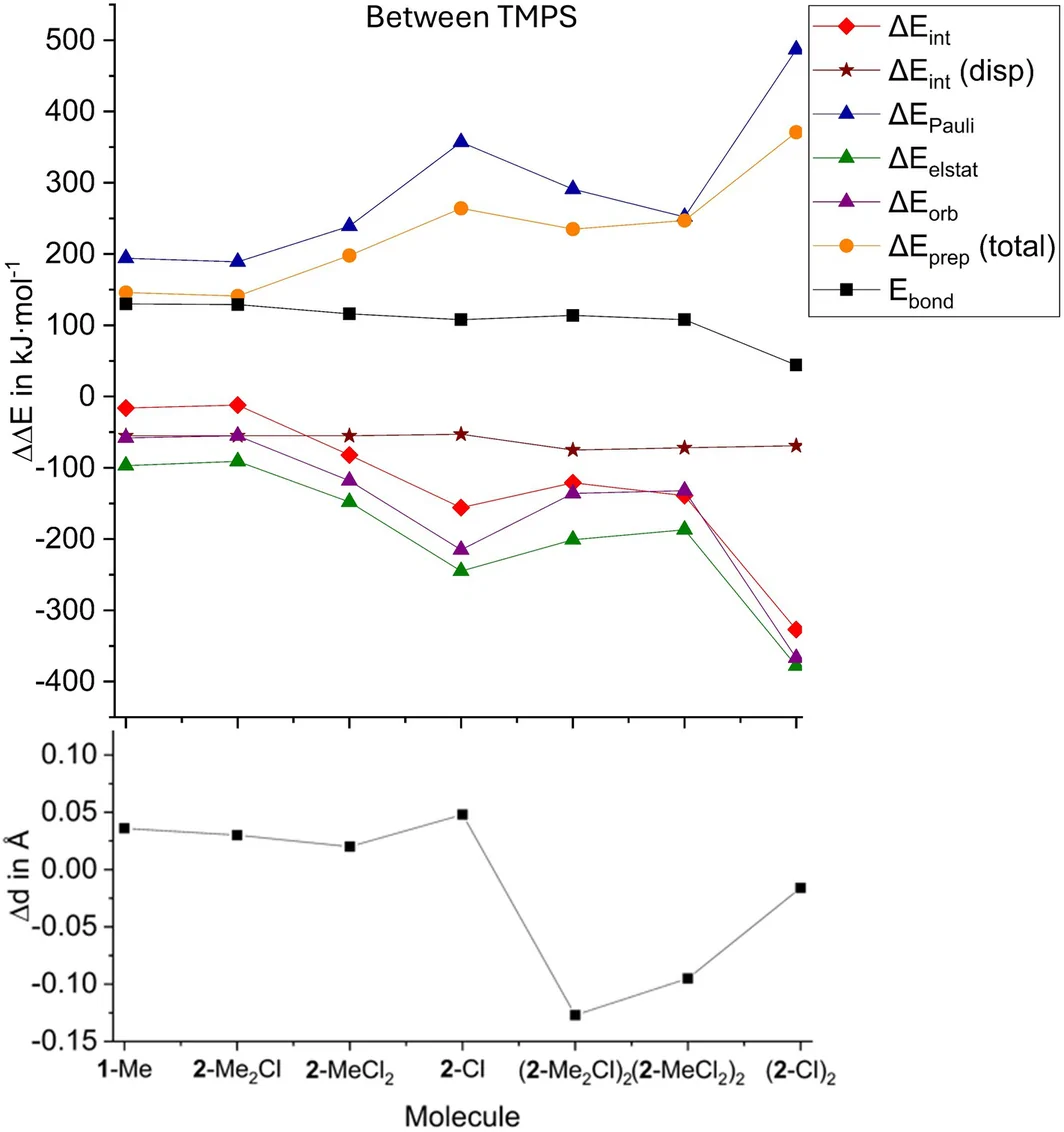
EDA terms of the precursors adsorbing between TMPS relative to the adsorption structures on SiO_2_. Bond length Δ*d* relative to the adsorption on free silica.

Table S17 shows the EDA terms of monomers and dimers adsorbing on SiO_2_ between TMPS while the EDA terms relative to the adsorption on the free SiO_2_ surface are shown in Figure 9.

As shown in Figure 9, $∆E_{\text{Pauli}}$ increases markedly for all structures relative to the free surface, rising by at least 189 kJ mol^−1^ (**2**‐MeCl_2_) and up to 487 kJ mol^−1^ for (**2**‐Cl)_2_. The $∆E_{\text{elstat}}$ and $∆E_{\mathrm{orb}}$ contributions increase across the series by 97 to 378 kJ mol^−1^ ($∆E_{\text{elstat}}$) and 58 to 367 kJ mol^−1^ ($∆E_{\mathrm{orb}}$), respectively. The $∆E_{\mathrm{int}}\left(\text{disp}\right)$ contributions also increases by 55 kJ mol^−1^ with **1**‐Me to 75 kJ mol^−1^ with (**2**‐MeCl_2_)_2_ relative to the free surface. $∆E_{\text{prep}}$ also increases substantially, rising by 141 kJ mol^−1^ from **2**‐MeCl_2_ to 371 kJ mol^−1^ for (**2**‐Cl)_2_. As a result of these combined effects $E_{\text{bond}}$ is significantly destabilized by 44 kJ mol^−1^ for (**2**‐Cl)_2_ to 130 kJ mol^−1^ for **1**‐Me. Bond lengths slightly increase for the monomers by up to 0.048 Å, whereas they decrease for the dimers by as much as 0.127 Å.

The much less stabilizing $E_{\text{bond}}$ shows that the surrounding SMIs exert an overall weakening effect on the precursor–surface bond, leading to energetically unfavorable bonding energies for nearly all precursors (e.g., a total $E_{\text{bond}}$ of 43 kJ mol^−1^ for the adsorption of **1**‐Me). The only exception is **2**‐Cl, for which adsorption remains favorable with respect to the separate precursor and surface, albeit much weaker (−34 kJ mol^−1^). However, for larger gap sizes within the inhibitor layer, it is conceivable that the attractive interactions—particularly the longer‐ranged electrostatic ($∆E_{\text{elstat}}$) and dispersion ($∆E_{\mathrm{int}}\left(\text{disp}\right)$) terms—could outweigh the short‐ranged Pauli repulsion and the $∆E_{\text{prep}}$ term. Under such conditions, the surrounding inhibitors might even favor precursor adsorption, a possibility that could be subject to further investigations.

While the trend with increasing Cl substitution is the same compared to the free surface (Figure S15a), the trend for the dimers differs markedly (Figure S15b). In contrast to the free surface, the dimers exhibit higher $∆E_{\text{elstat}}$ contributions than the monomers in all cases. Bond‐length analysis (Table S18) shows that the inhibitor layer forces the dimers to open, enhancing aluminum‐oxygen surface interactions compared to bare SiO_2_. Additional attractive interactions from the surrounding inhibitors further mitigate the destabilizing effect of dimerization, which is reduced or even reversed, as observed for (**2**‐Cl)_2_ which is more strongly bonded than **2**‐Cl despite its bulkiness.

In summary, the surrounding SMI layer reduces the adsorption strength of both monomers and dimers. This weakening arises primarily from steric repulsion and steric congestion, as reflected in the increased Pauli repulsion and deformation energy of the SMI layer, even though attractive interactions are simultaneously enhanced. These findings suggest that, at different scales, SMIs could potentially exert a favorable influence on adsorption—an avenue for further investigation in future studies. While dimer adsorption is likewise weakened, the effect is less pronounced compared to the free SiO_2_.

### 3.3 Design Principles for New Precursors

**FIGURE 10:**
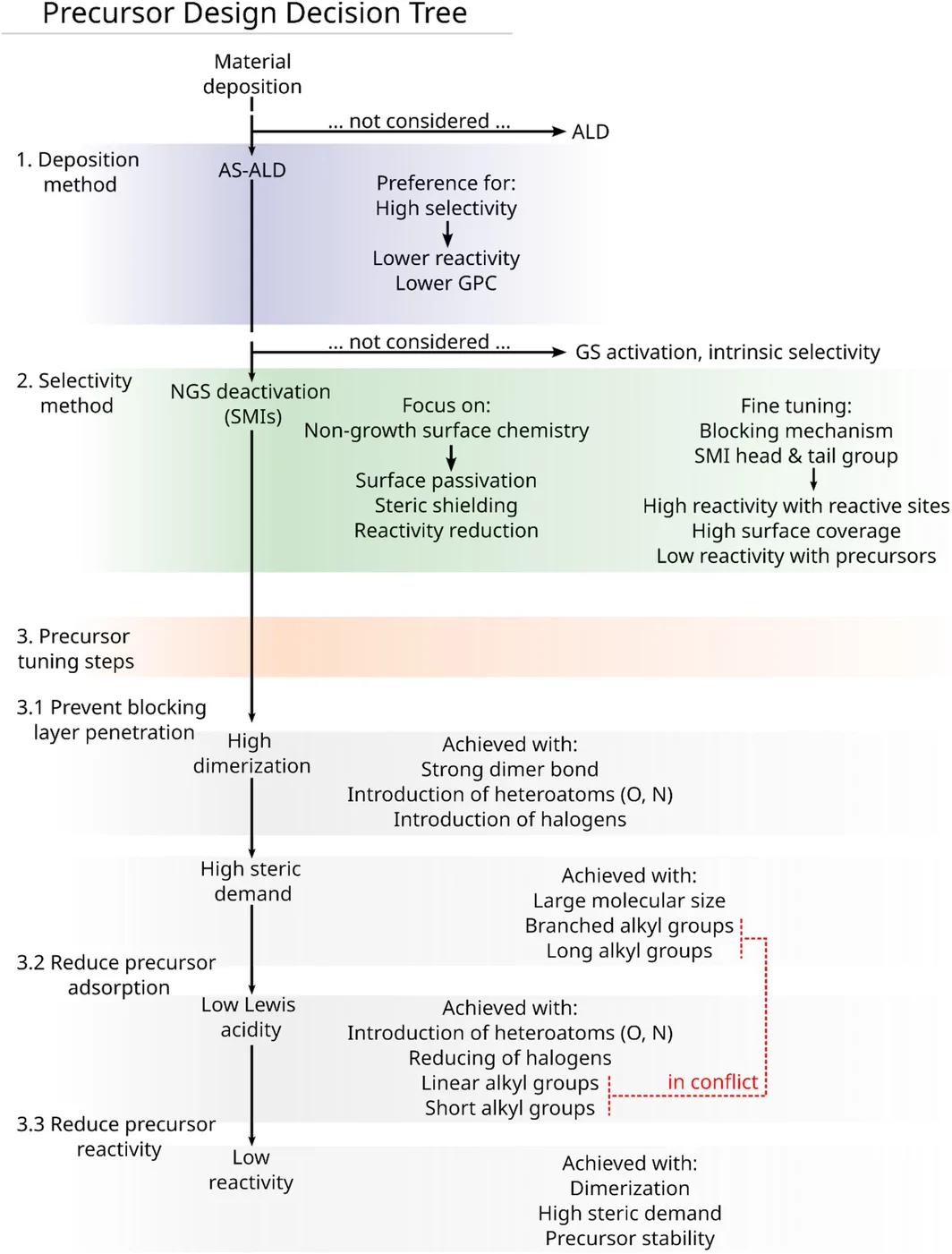
Systematic decision tree for the design of precursors based on our system.

We now examined the influence of precursor structure on bonding energies at the NGS in detail. However, to integrate these insights into a deeper understanding of selectivity in AS‐ALD and to guide the rational design of new precursors, it is essential to recognize that bonding energy represents only one aspect of surface reactivity. Additional factors must also be considered. By including the current literature addressing how precursor structure governs surface reactivity in ALD and AS‐ALD, we propose a design‐oriented decision tree. This framework, presented in Figure 10, integrates the key structure–reactivity relationships and provides a systematic basis for precursor design principles.

First and foremost, a fundamental distinction must be made as to whether the precursor is intended for conventional ALD or for AS‐ALD (Figure 10, Step 1). In ALD, high precursor reactivity is desirable to ensure efficient surface saturation and a high growth per cycle (GPC) [2]. In contrast, in AS‐ALD the preference is for high selectivity with the aim to suppress reactivity on the NGS, prioritizing selectivity over maximum growth efficiency at the GS. However, prioritizing selectivity likely comes with the inherent trade‐off of a lower overall GPC as for example shown by works of Oh et al. [7, 8] Conversely, precursors optimized for high growth rate in conventional ALD—such as **1**‐Me—may exhibit excessive reactivity on the NGS, rendering them unsuitable for AS‐ALD processes as it has often been found in the past [61].

The second key consideration concerns the strategy for achieving selectivity. In the following, we focus exclusively on the use of SMIs for NGS deactivation (Figure 10, Step 2). SMIs protect the surface through steric shielding and via chemical surface passivation of functional groups on the NGS; both effects have a crucial impact on selectivity [62]. Furthermore, the SMI layer may also reduce reactivity by increasing reaction barriers, as observed for SMI decomposition reactions [63]. These effects are influenced by the head and tail groups of the SMI, which can be chosen to achieve high reactivity with surface sites, high surface coverage, and reduced reactivity toward the precursors [11]. Alternative approaches to AS‐ALD—such as using self‐assembled monolayers (SAMs) as inhibitors [64], intrinsically selective processes [65, 66], or selective GS activation [67]—may involve different design criteria and are therefore not discussed here.

With the SMI approach the focus is the reactivity reduction on the NGS. To achieve this, precursor tuning steps (Figure 10, Step 3) are introduced. First, it is well established that minimizing the accessibility of reactive surface groups between the blocking layer is of central importance (Figure 10, Step 3.1) [12, 62, 63, 68]. As demonstrated previously as well as in the present work, precursor size strongly influences the ability to access surface sites located between inhibitors [6, 10, 11, 12]. Bulkier precursors have it more difficult to penetrate the SMI layer [12], although they may exhibit enhanced dispersion interactions.

Based on these considerations, we propose the following design principles for Al‐precursors: the highest priority is preventing the penetration of the blocking layer by targeting dimeric precursors. This increases the effective molecular size and reduces interaction with the NGS, as shown here and in previous studies [12, 14]. In the present system, dimerization can preferably be achieved by introducing an oxygen or nitrogen heteroatom, or alternatively by the introduction of halogens.

To further minimize penetration of the blocking layer, the steric demand of the precursor should be increased through appropriate substitution. This can be achieved by introducing sterically demanding groups, such as isopropyl or isobutyl substituents. In specific applications where heteroatom incorporation must be avoided, precursors free of oxygen, nitrogen, and chlorine are preferable. Under typical ALD conditions, such compounds would not undergo dimerization [14]. In this case, the emphasis would lie entirely on steric repulsion, with the introduction of bulky ligands serving to attenuate interactions with the NGS.

Next, once the accessibility to reactive groups has been reduced, further improvements in selectivity can be achieved by weakening the bonding to the NGS (Figure 10, Step 3.2). Weaker adsorption to the NGS or to the SMIs facilitates efficient precursor removal during the purge step and reduces the probability of reaction with residual surface species. As demonstrated here and in previous work [12], the likelihood of adsorption within the interstitial regions between SMIs is likewise affected by the bonding energy. As shown in this study, the incorporation of bulkier substituents such as isobutyl or isopropyl is advantageous not only for steric shielding but also for attenuating the binding strength to the NGS.

Another important factor influencing adsorption strength, as shown previously, is the Lewis acidity of the precursor. While the incorporation of oxygen or nitrogen as heteroatoms reduces Lewis acidity, chlorine substituents should be avoided. If chlorine is introduced to promote dimerization, the use of only a single chlorine substituent is recommended to limit the increase in Lewis acidity. This example illustrates that promoting dimerization and reducing Lewis acidity can be competing objectives, with dimerization generally taking priority. Alternative approaches to reducing Lewis acidity include the use of linear rather than branched short alkyl groups. However, this strategy is not compatible with the use of large, branched substituents to enhance steric repulsion and weaken adsorption, and is therefore not recommended.

Finally, after minimizing interactions with the NGS, the reactivity of the precursor remains as a parameter potentially influencing selectivity (Figure 10, Step 3.3). Due to the high reactivity of surface groups with aluminum precursors as shown by previous studies [7, 12, 63], we assume that avoiding adsorption to these groups is a more promising strategy than the reactivity reduction [63] of the precursor (i.e., the precursor reaches the surfaces but cannot react further due to steric constraints). Furthermore, the latter would negatively affect the GPC on the GS. Strategies to reduce precursor reactivity include promoting dimerization, increasing steric demand to shield the aluminum center, and selecting precursors with enhanced chemical stability through the introduction of heteroatoms. For example, calculations have shown that chlorinated aluminum precursors exhibit lower reactivity on SiO_2_ [7, 12]. In this way, the reduction in reactivity is consistent with most design strategies discussed above.

**FIGURE 11:**
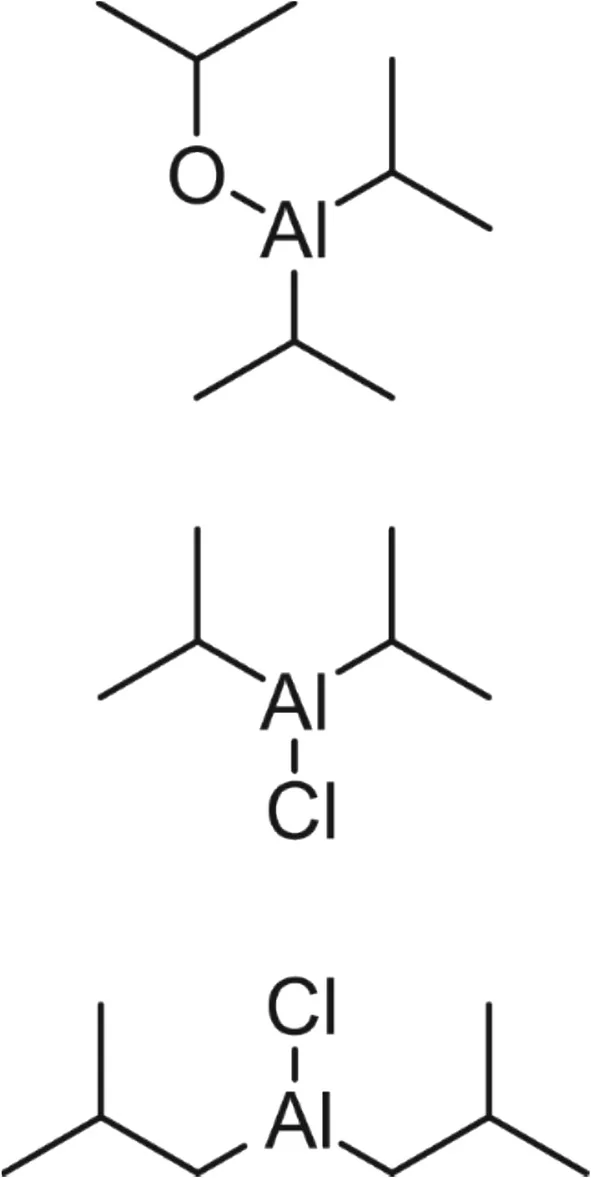
Proposed new aluminum precursors based on design principles outlined.

In summary, the scheme illustrates that certain objectives—most notably high GPC and high selectivity—can be inherently competing. However, it also highlights which of these targets may be reconciled through judicious ligand design. Accordingly, modification of the existing Al precursors depicted in Figure 1 is recommended to enhance their suitability for AS‐ALD. Promising strategies include substituting the methyl groups in DMAI with more sterically demanding ligands such as iPr, or employing chlorinated precursors containing only a single Cl substituent in combination with sterically bulky groups such as iPr or iBu to increase repulsive interactions and reduce undesired adsorption. Some of these recommended SMIs are shown in Figure 11.

## 4 Conclusions

We investigated how the substitution pattern of aluminum precursors affects their bonding characteristics in different environments: on free silica surface, on top of an inhibitor (TMPS), and in the gaps of a SMI layer. We thus focused on the relative importance of Lewis acidity, steric effects, and dimerization for the adsorption energies. The EDA and FIA approaches were to be checked for their suitability.

We show that Lewis acidity mostly provides a reliable predictor of trends in bonding energies. This holds for variations in alkyl chain length, the number of chlorine substituents, and the presence of heteroatoms O and N. In most cases, inductive and mesomeric effects, which are adequately captured by the FIA, govern the ordering of the bonding energies. In addition, dispersion represents a significant contribution that must be taken into account and, in the present systems, influences the trends in a manner consistent with Lewis acidity. An exception is observed for precursors containing sterically demanding branched alkyl groups (TiBA and TiPA), which exhibit weaker binding than expected from their FIA values, indicating that steric effects outweigh the influence of Lewis acidity. Another exception is TDMAA on SiO_2_, where proton transfer occurs and substantially enhances bonding to the surface. We conclude that the FIA will be a suitable tool for predicting the strength of precursor adsorption with limitations with bulky ligands or other unexpected effects like proton transfer.

Dimerization is another factor that strongly influences bonding on the NGS. In general, dimers are adsorbed weaker, and some—such as TDMAA and DMAI—do not open or form covalent bonds on the surface but are only weakly physisorbed. Others, such as the chlorinated dimers, undergo opening to varying degrees depending on the bonding environment. Overall, the additional interactions gained upon dimer opening are largely compensated by the additional dissociation energy.

Adsorption to the surface between the inhibitors represents a particularly critical scenario for AS‐ALD. Here, repulsion from neighboring groups and the deformation energy generally make adsorption unfavorable, despite the presence of additional attractive interactions. These are especially significant for dimers, and unlike adsorption on free SiO_2_ or on top of an SMI, the weakening effect of dimers on adsorption may be reduced—or even reversed—within the inhibitor layer. Lewis acidity enhances the likelihood of adsorption between inhibitors. Although SMIs disfavor adsorption in our system, it is plausible that under certain conditions the attractive interactions could prevail.

By combining the knowledge of the underlying mechanisms resulting in selectivity loss in AS‐ALD and the influence of substituents on the adsorption characteristics of precursors we propose a systematic strategy to design precursors for AS‐ALD. Although this study is limited to Al precursors, these insights can be transferred to other (Lewis‐acidic) precursors, for example, for B, Ga or Zn.

## Funding

This work was supported by 350th Anniversary Grant (Merck KGaA, Darmstadt, Germany).

## Conflicts of Interest

The authors declare no conflicts of interest.

## Supporting information


**Section 1:** Testing k‐grid for EDA calculations.
**Table S1:** k‐grid convergence in AMS‐BAND.^[a]^
**Section 2:** Structures.
**Figure S1:** Illustration of the concept of preparation energy. Figure reproduced from Maue et al. [1] 2026, licensed under CC BY 4.0.
**Figure S2:** Proton transfer to **3** on the SiO_2_ surface. Bond length in Å.
**Section 3:** Orbital overlap.
**Table S2:** Orbital overlap between the LUMO of the precursor and the overlapping p‐orbital of the F^−^ ion.
**Section 4:** EDA on Methanol.
**Figure S3:** Absolute energy terms of the EDA of **1**‐Me and the chlorinated precursors of group **2** adsorbing to Methanol and TMPS as comparison.
**Figure S4:** Absolute energy terms of the EDA of the dimers of the chlorinated precursors adsorbing to Methanol and TMPS as comparison.
**Figure S5:** Absolute energy terms of the EDA of the alkyl substituted precursors of group **1** adsorbing to Methanol and TMPS as comparison.
**Figure S6:** Absolute energy terms of the EDA of **1**‐Me, **3** and **4** adsorbing to methanol and TMPS as comparison.
**Section 5:** EDA on SiO_2_.
**Table S3:** EDA terms of chlorinated precursors on SiO_2_.^[a]^
**Table S4:** Hirshfeld charges of the aluminum atom dependent on the number of Cl for the precursors **1**‐Me to **2**‐Cl in gas phase.
**Table S5:** EDA terms of alkyl‐substituted precursors **1** on SiO_2_.^[a]^
**Table S6:** EDA of **1**‐Me and **4** on SiO_2_.^[a]^
**Section 6:** EDA on TMPS.
**Figure S7:** pEDA terms relative to **1**‐Me for the alkyl substituted precursors **1** on (a) SiO_2_ (Figure 2a) and (b) TMPS on SiO_2_ (Figure 2b) with the original conformers.
**Figure S8:** (a) **1**‐iPr on SiO_2_. (b) **1**‐iBu on SiO_2_. (c) **1**‐iPr on TMPS. (d) **1**‐iBu on TMPS.
**Table S7:** Conformer sampling of **1**‐iBu.^[a]^
**Table S8:** Conformer sampling of **1**‐iPr.^[a]^
**Table S9:** EDA terms of the chlorinated precursors on TMPS.^[a]^
**Table S10:** EDA terms of the alkyl substituted precursors on TMPS.^[a]^
**Table S11:** EDA of **1**‐Me, **3** and **4** on TMPS.^[a]^
**Section 7:** FIA vs. EDA terms.
**Figure S9:** (a) Negative Δ*E*_int_ versus FIA for the tested set of precursors adsorbing to SiO_2_. The outliers **1**‐iPr and **1**‐iBu are marked in red. (b) Negative Δ*E*_int_ versus FIA for the tested set of precursors adsorbing to TMPS on SiO_2_.
**Figure S10:** (a) Δ*E*_Pauli_ versus FIA for the tested set of precursors adsorbing on SiO_2_. The outliers **1**‐iPr and **1**‐iBu are marked in red. (b) Δ*E*_Pauli_ versus FIA for the tested set of precursors adsorbing to TMPS on SiO_2_.
**Figure S11:** (a) Δ*E*_elstat_ versus FIA for the tested set of precursors adsorbing on SiO_2_. (b) Δ*E*_elstat_ versus FIA for the tested set of precursors adsorbing on TMPS.
**Figure S12:** (a) Δ*E*_orb_ versus FIA for the tested set of precursors adsorbing on SiO_2_. (b) Δ*E*_orb_ versus FIA for the tested set of precursors adsorbing to TMPS on SiO_2_.
**Table S12:** Fit parameters of the EDA vs. FIA values for the adsorption to SiO_2_.
**Table S13:** Fit parameters of the EDA vs. FIA values for the adsorption to the methoxy group of TMPS.
**Section 8:** EDA of dimers.
**Table S14:** EDA terms of dimers compared to monomers on SiO_2_.^[a]^
**Table S15:** EDA terms of dimers compared to monomers on TMPS on SiO_2_.^[a]^
**Table S16:** Bond length comparison of the broken Al‐Cl dimer bond for the adsorption of the dimers to SiO_2_ and to the methoxy group of TMPS.^[a]^
**Section 9:** EDA of precursors adsorbing in between SMI‐layer.
**Figure S13:** Adsorption structures and energies of the monomers that can adsorb between the TMPS layer. Dispersion contribution in brackets. Figure reproduced from Maue et al. [7] 2026, licensed under CC BY 4.0.
**Figure S14:** Adsorption structures and energies of the chlorinated dimers that can adsorb between the TMPS layer. Dispersion contribution in brackets. Figure reproduced from Maue et al. [7] 2026, licensed under CC BY 4.0.
**Table S17:** EDA of monomers and dimers adsorbing between TMPS.^[a]^
**Figure S15:** (a) pEDA terms of monomers of group **2** relative to **1**‐Me adsorbing to the surface between TMPS. (b) pEDA terms of the dimers of group **2** relative to their corresponding monomers with the difference in bond distance between dimers and monomers Δd.
**Table S18:** Bond length comparison of the broken Al‐Cl dimer bond for the adsorption of the dimers on free SiO_2_ and on SiO_2_ between the inhibitors.^[a]^

## Data Availability

All computational data are available in the open access database Zenodo via https://doi.org/10.5281/zenodo.21703627.
