# Supplementary material for: Precursor Design Principles for Area‐Selective Atomic Layer Deposition of Alumina: Quantifying the Role of Lewis Acidity, Steric Repulsion, and Dimerization in Surface Adsorption
Source: J Comput Chem. 2026 Sep 3;47(23):e70493. doi: 10.1002/jcc.70493 (PMC13539961; doi:10.1002/jcc.70493)
Supplement: Supplementary file 1 [file JCC-47-0-s001.docx]

**Precursor design principles for area-selective atomic layer deposition of alumina: quantifying the role of Lewis acidity, steric repulsion and dimerization in surface adsorption**

**Patrick Maue, Douglas L. Miller, Fabian Pieck, Ralf Tonner-Zech^[a],*^**

**Table of Contents**

1. **Testing k-grid for EDA calculations**	**2**
2. **Structures**	**2**
3. **Orbital overlap**	**3**
4. **EDA on Methanol**	**3**
5. **EDA on SiO_2_**	**8**
6. **EDA on TMPS**	**9**
7. **FIA vs EDA terms**	**14**
8. **EDA of dimers**	**17**
9. **EDA of precursors adsorbing between TMPS**	**19**

[a] Patrick Maue, Douglas Miller, Dr. Fabian Pieck, Prof. Dr. R. Tonner-Zech

Fakultät für Chemie, Wilhelm-Ostwald-Institut für Physikalische und Theoretische Chemie, Universität Leipzig, Linnéstr. 2, 04103 Leipzig, Germany

e-mail: [ralf.tonner@uni-leipzig.de](mailto:ralf.tonner@uni-leipzig.de)

ORCID: Patrick Maue 0009-0009-1183-0775, Doug Miller 0009-0005-6606-903X, Fabian Pieck 0000-0001-6912-2725, Ralf Tonner-Zech 0000-0002-6759-8559

1. **Testing k-grid for EDA calculations**

To assess whether a Γ-only k-point sampling in AMS-BAND is sufficient, we compared it with a denser k-point grid for the adsorption of **1**-Me on SiO_2_. Table S1 summarizes the EDA terms obtained using Γ-only sampling and the “good” k-grid setting in AMS-BAND, which corresponds to a (3 × 3) k-point grid for the present system.

**Table S1.** k-grid convergence in AMS-BAND.^[a]^

| SiO_2_ | **1**-Me (Γ-only) | **1**-Me (3x3) |
| --- | --- | --- |
| ΔE_int_ | -144.7 | -144.8 |
| ΔE_int_ (disp) | -51.5 | -51.5 |
| ΔE_int_ (elec) | -93.2 | -93.3 |
| ΔE_Pauli_ | 283.5 | 283.5 |
| ΔE_elstat_ | -232.2 | -232.2 |
| ΔE_orb_ | -144.5 | -144.6 |
| ΔE_prep_ (mol) | 26.6 | 26.6 |
| ΔE_prep_ (surface) | 31.5 | 31.5 |
| E_bond_ | -86.6 | -86.7 |

[a] Energies in kJ·mol^-1^. EDA of **1**-Me adsorbing to SiO_2_ calculated with Γ-only (left) compared to a 3x3 k-grid (parameter “good”) in AMS-BAND (right).

The result show deviations of <1 kJ·mol^-1^ and therefore support the use of Γ-only settings in all calculations.

2. **Structures**

**Figure S1.** Illustration of the concept of preparation energy. Figure reproduced from Maue et al.,^1^ 2026, licensed under CC BY 4.0.

**Figure S2.** Proton transfer to **3** on the SiO_2_ surface. Bond length in Å.

3. **Orbital overlap**

The orbital overlap was calculated using PyFrag 2019.^2^ based on single-point calculations of the molecular fragments performed with AMS 2023.101.^3^ The PBE0^4^ functional was employed in conjunction with the TZ2P basis set.^5,6^ The numerical integration quality was set to “very good”. The SCF convergence criterion was set to 10^-6^ Eh, and no frozen-core approximation was applied. Table S2 shows the result with the corresponding bond lengths.

**Table S2.** Orbital overlap between the LUMO of the precursor and the overlapping p-orbital of the F^-^ ion

|  | **2**-Cl | **2**-MeCl_2_ | **2**-Me_2_Cl | **4** | **3** | **1**-Me | **1**-Et | **1**-Pr | **1**-iPr | **1**-iBu |
| --- | --- | --- | --- | --- | --- | --- | --- | --- | --- | --- |
| Overlap | 0.220 | 0.183 | 0.168 | 0.161 | 0.162 | 0.164 | 0.154 | 0.143 | 0.147 | 0.148 |
| d Al-F^-[a]^ | 1.672 | 1.688 | 1.705 | 1.717 | 1.705 | 1.724 | 1.722 | 1.721 | 1.721 | 1.723 |

[a] Bond length in Å

4. **EDA on Methanol**

To evaluate the influence of the surface on the observed trends in the EDA terms, additional bonding analyses were performed for precursor adsorption to methanol. The resulting trends were compared with those obtained for adsorption to the methoxy group of TMPS (see Figure 2b in the main text). For precursors where reactivity is not governed solely by Lewis acidity, differences between the two bonding scenarios might be anticipated. Figure S3 presents the evolution of the EDA contributions with increasing number of chlorine substituents for adsorption to methanol, in comparison to the methoxy group of TMPS.

**Figure S3.** Absolute energy terms of the EDA of **1**-Me and the chlorinated precursors of group **2** adsorbing to Methanol and TMPS as comparison.

As expected, the trend in $E_{\mathrm{bond}}$, which follows the order of Lewis acidity, remains unchanged upon adsorption to methanol for this set of precursors. Only quantitative differences are observed. These can be attributed to the larger number of atoms involved in the interaction during adsorption to TMPS, as well as to the distinct chemical environment of the methoxy oxygen. Because the silicon atom in TMPS is less electronegative than the hydrogen atom in methanol, the oxygen atom in TMPS is expected to exhibit higher electron density and stronger donor ability. This results in more favorable $\Delta E_{\mathrm{elstat}}$ and $\Delta E_{\mathrm{orb}}$ contributions, leading to a stronger $E_{\mathrm{bond}}$, further enhanced by increased dispersion contributions. The adsorption to methanol instead of TMPS leads to more pronounced differences when dimers are involved as the EDA terms in Figure S4 show for both bonding situations.

**Figure S4.** Absolute energy terms of the EDA of the dimers of the chlorinated precursors adsorbing to Methanol and TMPS as comparison.

For the dimers, the differences in the individual EDA contributions between TMPS and methanol largely compensate each other, such that $E_{\mathrm{bond}}$ differs by less than 10 kJ·mol^-1^ despite more pronounced variations in the separate terms. This behavior contrasts with that of the other precursors, which generally form stronger bonds to TMPS than to methanol in the gas phase. The distinct behavior of the dimers can be attributed to their higher $\Delta E_{\mathrm{prep}}$, as greater structural deformation is required to reach the bonding geometry compared to the monomeric species. In this case, adsorption to TMPS induces larger changes than for the monomers, preventing the expected decrease in $E_{\mathrm{bond}}$. In case of the adsorption of the alkyl substituted precursors of class **1**, the chemical trends are similar as Figure S5 shows.

**Figure S5.** Absolute energy terms of the EDA of the alkyl substituted precursors of group **1** adsorbing to Methanol and TMPS as comparison.

The ${\Delta E}_{\mathrm{int}}\left( \mathrm{disp} \right)$, $\Delta E_{\mathrm{Pauli}}$, $\Delta E_{\mathrm{elstat}}$, and $\Delta E_{\mathrm{orb}}$ contributions are consistently larger for adsorption to TMPS than to methanol. $E_{\mathrm{prep}}$ is likewise increased, except for **1**-iBu. Overall, these effects result in a stronger $E_{\mathrm{bond}}$ while preserving the same chemical trends with respect to alkyl chain length and branching. For precursors **3** and **4**, comparable trends are observed for adsorption to methanol in comparison to TMPS, as illustrated in Figure S6.

**Figure S6.** Absolute energy terms of the EDA of **1**-Me, **3** and **4** adsorbing to methanol and TMPS as comparison.

In summary, adsorption to TMPS versus methanol primarily leads to quantitative differences in the bonding contributions. In most cases, all terms increase, with the enhancement of the attractive interactions ($\Delta E_{\mathrm{elstat}}$ and $\Delta E_{\mathrm{orb}}$) outweighing the increases in $\Delta E_{\mathrm{Pauli}}$ and $\Delta E_{\mathrm{prep}}$, except for the dimers of the chlorinated precursors. Overall, the chemical trends are preserved, with only a few exceptions.

5. **EDA on SiO_2_**

**Table S3.** EDA terms of chlorinated precursors on SiO_2_.^[a]^

|  | **1**-Me | **2**-Me_2_Cl | **2**-MeCl_2_ | **2**-Cl |
| --- | --- | --- | --- | --- |
| ΔE_int_ | -145 | -177 | -213 | -239 |
| ΔE_int_ (disp) | -52 (36%) | -51 (29%) | -49 (23%) | -49 (21%) |
| ΔE_int_ (elec) | -93 (64%) | -126 (71%) | -164 (77%) | -190 (79%) |
| ΔE_Pauli_ | 282 | 324 | 373 | 391 |
| ΔE_elstat_ | -231 (62%) | -276 (61%) | -323 (60%) | -344 (59%) |
| ΔE_orb_ | -144 (38%) | -174 (39%) | -214 (40%) | -237 (41%) |
| ΔE_prep_ (mol) | 26 | 28 | 35 | 42 |
| ΔE_prep_ (surf) | 32 | 47 | 49 | 55 |
| ΔE_prep_ (total) | 58 | 75 | 84 | 97 |
| E_bond_ (PAW)^[b]^ | -87 (-90) | -103 (-106) | -128 (-134) | -142 (-148) |
| R (O-Al) | 2.024 | 1.982 | 1.930 | 1.891 |

[a] Energies in kJ∙mol^-1^, bond lengths in Å

[b] Energies calculated in VASP with a plane wave basis are shown in parentheses. Values for **1**-Me and **2**-Cl are already published in ref.^1^

To better understand the trends of the bonding energies, charges were calculated for the precursors for the chlorinated precursors, shown in the following table.

**Table S4.** Hirshfeld charges of the aluminum atom dependent on the number of Cl for the precursors **1**-Me to **2**-Cl in gas phase.

| Hirshfeld | Al |
| --- | --- |
| **1**-Me | 0.501 |
| **2**-Me_2_Cl | 0.503 |
| **2**-MeCl_2_ | 0.504 |
| **2**-Cl | 0.511 |

**Table S5.** EDA terms of alkyl-substituted precursors **1** on SiO_2_.^[a]^

|  | **1**-Me | **1**-Et | **1**-Pr | **1**-iPr | **1**-iBu |
| --- | --- | --- | --- | --- | --- |
| ΔE_int_ | -145 | -154 | -171 | -161 | -144 |
| ΔE_int_ (disp) | -52 (36%) | -66 (43%) | -86 (50%) | -88 (55%) | -84 (58%) |
| ΔE_int_ (elec) | -93 (64%) | -88 (57%) | -85 (50%) | -74 (45%) | -60 (42%) |
| ΔE_Pauli_ | 282 | 296 | 315 | 300 | 258 |
| ΔE_elstat_ | -231 (62%) | -237 (62%) | -248 (62%) | -228 (61%) | -194 (61%) |
| ΔE_orb_ | -144 (38%) | -147 (38%) | -152 (38%) | -146 (39%) | -124 (39%) |
| ΔE_prep_ (mol) | 26 | 27 | 31 | 31 | 24 |
| ΔE_prep_ (surf) | 32 | 31 | 31 | 50 | 50 |
| ΔE_prep_ (total) | 58 | 58 | 62 | 81 | 73 |
| E_bond_ (PAW)^[b]^ | -87 (-90) | -96 (-99) | -109 (-113) | -80 (-100) | -71 (-89) |
| R (O-Al) | 2.024 | 2.031 | 2.024 | 2.093 | 2.144 |

[a] Energies in kJ·mol^-1^, bond lengths in Å

[b] Energies calculated in VASP with a plane wave basis are shown in parentheses. Value for **1**-Me is already published in ref.^1^

**Table S6.** EDA of **1**-Me and **4** on SiO_2_.^[a]^

|  | **1**-Me | **4** |
| --- | --- | --- |
| ΔE_int_ | -145 | -160 |
| ΔE_int_ (disp) | -52 (36%) | -69 (43%) |
| ΔE_int_ (elec) | -93 (64%) | -91 (57%) |
| ΔE_Pauli_ | 282 | 307 |
| ΔE_elstat_ | -231 (62%) | -246 (62%) |
| ΔE_orb_ | -144 (38%) | -152 (38%) |
| ΔE_prep_ (mol) | 26 | 41 |
| ΔE_prep_ (surf) | 32 | 33 |
| ΔE_prep_ (total) | 58 | 74 |
| E_bond_ (PAW)^[b]^ | -87 (-90) | -87 (-90) |
| R (O-Al) | 2.024 | 2.012 |

[a] Energies in kJ∙mol^-1^, bond lengths in Å

[b] Energies calculated in VASP with a plane wave basis are shown in parentheses. Both values are already published in ref.^1^

6. **EDA on TMPS**

This section presents the EDA tables for the adsorption of the different precursor classes to the methoxy group of TMPS (see Figure 2b in the main text). Due to the unexpected results obtained for the precursors bearing branched alkyl groups, and the influence of alkyl-group orientation on, for example, dispersion interactions (Figures S7 and S8), a conformer search was performed for **1**-iPr and **1**-iBu. The corresponding results are summarized in Tables S7 and S8.

**Figure S7.** pEDA terms relative to **1**-Me for the alkyl substituted precursors **1** on (a) SiO_2_ (Figure 2a) and (b) TMPS on SiO_2_ (Figure 2b) with the original conformers.

**Figure S8.** (a) **1**-iPr on SiO_2_. (b) **1**-iBu on SiO_2_. (c) **1**-iPr on TMPS. (d) **1**-iBu on TMPS.

**Table S7.** Conformer sampling of **1**-iBu.^[a]^

|  | ΔE_ads_ (SiO_2_) | d in Å (SiO_2_) | ΔE_ads_ (TMPS) | d in Å (TMPS) |
| --- | --- | --- | --- | --- |
| **TiBA_original** | -79 | 2.170 | **-93** | 2.130 |
| TiBA_conf1 | -77 | 2.165 | -80 | 2.162 |
| TiBA_conf2 | -85 | 2.139 | -61 | 2.186 |
| TiBA_conf3 | -78 | 2.153 | -81 | 2.164 |
| TiBA_conf4 | -86 | 2.142 | -85 | 2.134 |
| TiBA_conf5 | -77 | 2.159 | -80 | 2.196 |
| TiBA_conf6 | -77 | 2.163 | -74 | 2.154 |
| **TiBA_conf7** | **-89** | 2.144 | -78 | 2.172 |
| TiBA_conf8 | -85 | 2.145 | -84 | 2.151 |
| TiBA_conf9 | -74 | 2.147 | -70 | 2.200 |
| TiBA_conf10 | -75 | 2.143 | -69 | 2.233 |

[a] All energies in kJ·mol^-1^, bond lengths d in Å. Selected conformers for the EDA are highlighted in bold.

**Table S8.** Conformer sampling of **1**-iPr.^[a]^

|  | ΔE_ads_ (SiO_2_) | d in Å (SiO_2_) | ΔE_ads_ (TMPS) | d in Å (TMPS) |
| --- | --- | --- | --- | --- |
| TiPA_original | -88 | 2.136 | -79 | 2.091 |
| TiPA_conf1 | -95 | 2.123 | -85 | 2.171 |
| TiPA_conf2 | -89 | 2.103 | -82 | 2.125 |
| TiPA_conf3 | -95 | 2.116 | -86 | 2.112 |
| **TiPA_conf4** | **-100** | 2.093 | -78 | 2.155 |
| TiPA_conf5 | -93 | 2.139 | -90 | 2.113 |
| TiPA_conf6 | -92 | 2.094 | -85 | 2.113 |
| TiPA_conf7 | -93 | 2.138 | -90 | 2.119 |
| TiPA_conf8 | -96 | 2.123 | -82 | 2.119 |
| TiPA_conf9 | -100 | 2.109 | -88 | 2.097 |
| **TiPA_conf10** | -97 | 2.088 | **-92** | 2.109 |

[a] All energies in kJ·mol^-1^, bond lengths d in Å. Selected conformers for the EDA are highlighted in bold.

Table S9 reports the EDA results for **1**-Me and the series of chlorinated precursors adsorbed on the methoxy group of TMPS.

**Table S9.** EDA terms of the chlorinated precursors on TMPS.^[a]^

|  | **1**-Me | **2**-Me_2_Cl | **2**-MeCl_2_ | **2**-Cl |
| --- | --- | --- | --- | --- |
| ΔE_int_ | -121 | -143 | -173 | -204 |
| ΔE_int_ (disp) | -47 (39%) | -48 (34%) | -47 (27%) | -48 (24%) |
| ΔE_int_ (elec) | -74 (61%) | -95 (66%) | -126 (73%) | -156 (76%) |
| ΔE_Pauli_ | 238 | 288 | 330 | 353 |
| ΔE_elstat_ | -198 (63%) | -238 (62%) | -280 (61%) | -306 (60%) |
| ΔE_orb_ | -114 (37%) | -145 (38%) | -176 (39%) | -203 (40%) |
| ΔE_prep_ (mol) | 23 | 29 | 33 | 39 |
| ΔE_prep_ (surf) | 11 | 16 | 22 | 25 |
| ΔE_prep_ (total) | 34 | 45 | 55 | 64 |
| E_bond_ (PAW)^[b]^ | -87 (-82) | -98 (-95) | -118 (-116) | -140 (-138) |
| R (O-Al) | 2.074 | 2.015 | 1.966 | 1.927 |

[a] Energies in kJ∙mol^-1^, bond lengths in Å

[b] Energies calculated in VASP with a plane wave basis are shown in parentheses

The chemical trends are consistent with those observed on the SiO_2_ surface: precursors bind more strongly with increasing numbers of chlorine substituents, and each Cl atom contributes a ${\Delta E}_{\mathrm{int}}\left( \mathrm{disp} \right)$ comparable to that of a methyl group. The increases in attractive contributions, $\Delta E_{\mathrm{elstat}}$ and $\Delta E_{\mathrm{orb}}$, outweigh the corresponding increases in the destabilizing terms, resulting in stronger overall bonding. The EDA results for the adsorption of various alkyl-substituted class **1** precursors to TMPS with the selected conformers are summarized in Table S10.

**Table S10.** EDA terms of the alkyl substituted precursors on TMPS.^[a]^

|  | **1**-Me | **1**-Et | **1**-Pr | **1**-iPr | **1**-iBu |
| --- | --- | --- | --- | --- | --- |
| ΔE_int_ | -121 | -125 | -138 | -138 | -144 |
| ΔE_int_ (disp) | -47 (39%) | -61 (49%) | -75 (54%) | -78 (57%) | -86 (60%) |
| ΔE_int_ (elec) | -74 (61%) | -64 (51%) | -63 (46%) | -60 (43%) | -58 (40%) |
| ΔE_Pauli_ | 238 | 254 | 262 | 264 | 259 |
| ΔE_elstat_ | -198 (63%) | -201 (63%) | -206 (63%) | -203 (63%) | -200 (63%) |
| ΔE_orb_ | -114 (37%) | -117 (37%) | -119 (37%) | -121 (37%) | -117 (37%) |
| ΔE_prep_ (mol) | 23 | 25 | 24 | 28 | 32 |
| ΔE_prep_ (surf) | 11 | 13 | 12 | 33 | 13 |
| ΔE_prep_ (total) | 34 | 38 | 36 | 60 | 45 |
| E_bond_ (PAW)^[b]^ | -87 (-82) | -87 (-85) | -102 (-98) | -78 (-92) | -99 (-93) |
| R (O-Al) | 2.074 | 2.093 | 2.088 | 2.109 | 2.130 |

[a] Energies in kJ·mol^-1^, bond lengths in Å

[b] Energies calculated in VASP with a plane wave basis are shown in parentheses

The trend of increasing $E_{\mathrm{bond}}$ with alkyl chain length and the destabilization associated with branched alkyl groups of comparable size is also observed for adsorption to TMPS. Attractive contributions grow with chain length, enhancing $\Delta E_{\mathrm{int}}$, like the behavior on pristine SiO_2_. However, the effect of branched groups causing longer bond distances and reduced electrostatic and orbital interactions compared to linear analogues is not apparent for TMPS adsorption. This can be rationalized by the reduced steric repulsion experienced by branched groups when interacting with a methoxy group compared to the surface. Additionally, the $\Delta E_{\mathrm{prep}}$ values for TMPS adsorption are generally lower than those for adsorption on pristine SiO_2_.

The EDA for the adsorption of the precursors with **3** and **4** to TMPS compared to **1**-Me is shown in Table S11.

**Table S11.** EDA of **1**-Me, **3** and **4** on TMPS.^[a]^

|  | **1**-Me | **3** | **4** |
| --- | --- | --- | --- |
| ΔE_int_ | -121 | -141 | -127 |
| ΔE_int_ (disp) | -47 (39%) | -77 (55%) | -51 (40%) |
| ΔE_int_ (elec) | -74 (61%) | -64 (45%) | -76 (60%) |
| ΔE_Pauli_ | 238 | 329 | 278 |
| ΔE_elstat_ | -198 (63%) | -254 (65%) | -226 (64%) |
| ΔE_orb_ | -114 (37%) | -139 (35%) | -128 (36%) |
| ΔE_prep_ (mol) | 23 | 39 | 27 |
| ΔE_prep_ (surf) | 11 | 20 | 11 |
| ΔE_prep_ (total) | 34 | 59 | 38 |
| E_bond_ (PAW)^[b]^ | -87 (-82) | -82 (-77) | -89 (-86) |
| R (O-Al) | 2.074 | 2.085 | 2.049 |

[a] Energies in kJ∙mol^-1^, bond lengths in Å

[b] Energies calculated in VASP with a plane wave basis are shown in parentheses

As on pristine SiO_2_, **1**-Me and **4** exhibit very similar $E_{\mathrm{bond}}$ values, differing by only 2 kJ·mol^-1^. Precursor **3** is bulkier, resulting in a longer bond length than **1**-Me, whereas **4** is smaller but binds more strongly at a shorter distance. The order of $E_{\mathrm{bond}}$ corresponds to the order of FIAs for these precursors. $\Delta E_{\mathrm{prep}}$ increases with precursor size and governs the $E_{\mathrm{bond}}$ trend, as $\Delta E_{\mathrm{int}}$ follows a different pattern. The ${\Delta E}_{\mathrm{int}}\left( \mathrm{disp} \right)$ contribution also grows with size, and the overall trend in $\Delta E_{\mathrm{int}}$ is dominated by attractive interactions rather than by $\Delta E_{\mathrm{Pauli}}$.

7. **FIA vs EDA terms**

Figure S9 to S12 show the correlation between the individual EDA terms including $\Delta E_{\mathrm{int}}$, $\Delta E_{\mathrm{Pauli}}$, $\Delta E_{\mathrm{elstat}}$ and $\Delta E_{\mathrm{orb}}$ with the FIA for the precursors adsorbing to SiO_2_ and TMPS on SiO_2_.

**Figure S9.** (a) Negative Δ*E*_int_ versus FIA for the tested set of precursors adsorbing to SiO_2_. The outliers **1**-iPr and **1**-iBu are marked in red. (b) Negative Δ*E*_int_ versus FIA for the tested set of precursors adsorbing to TMPS on SiO_2_.

The trend observed for $\Delta E_{\mathrm{int}}$ is like that of $E_{\mathrm{bond}}$. However, the linear correlation improves, with an R² value of 0.90 for adsorption to SiO_2_ and to TMPS when all precursors are included. On SiO_2_, **1**-iPr and **1**-iBu remain outliers, and the R^2^ increases to 0.96 when these molecules are excluded. For TMPS adsorption, deviations of **1**-Pr and **1**-iBu from the trend are smaller, consistent with the reduced steric repulsion provided by the methoxy group. Precursor **3** exhibits a weaker $\Delta E_{\mathrm{int}}$ relative to the overall trend, but this is offset by a higher $\Delta E_{\mathrm{prep}}$, resulting in an $E_{\mathrm{bond}}$ that aligns with the general trend (see Figure 6 in the main text).

**Figure S10.** (a) Δ*E*_Pauli_ versus FIA for the tested set of precursors adsorbing on SiO_2._ The outliers **1**-iPr and **1**-iBu are marked in red. (b) Δ*E*_Pauli_ versus FIA for the tested set of precursors adsorbing to TMPS on SiO_2_.

For the $\Delta E_{\mathrm{Pauli}}$ term, the R^2^ value for adsorption to SiO_2_ is 0.77 when all precursors are included and improves to 0.94 when **1**-iPr and **1**-iBu are excluded. For adsorption to the methoxy group of TMPS, the R^2^ value is 0.49. The weaker correlation is primarily due to precursor **3**, which exhibits a significantly higher $\Delta E_{\mathrm{Pauli}}$ than would be expected based on its FIA.

**Figure S11.** (a) Δ*E*_elstat_ versus FIA for the tested set of precursors adsorbing on SiO_2_. (b) Δ*E*_elstat_ versus FIA for the tested set of precursors adsorbing on TMPS.

For $\Delta E_{\mathrm{elstat}}$, the R^2^ value is 0.77 when all precursors are included and increases to 0.97 when **1**-iPr and **1**-iBu are excluded. For adsorption to the methoxy group of TMPS, the R^2^ value is 0.64. The lower correlation in this case is primarily due to the unusually small $\Delta E_{\mathrm{elstat}}$ contribution of precursor **3**.

**Figure S12.** (a) Δ*E*_orb_ versus FIA for the tested set of precursors adsorbing on SiO_2_. (b) Δ*E*_orb_ versus FIA for the tested set of precursors adsorbing to TMPS on SiO_2_.

For $\Delta E_{\mathrm{orb}}$, the R^2^ value is 0.86 when all precursors are included and increases to 0.98 when **1**-iPr and **1**-iBu are excluded. For adsorption to the methoxy group of TMPS, the R^2^ value is 0.84. The correlation is improved for TMPS because precursor **3** deviates less for $\Delta E_{\mathrm{orb}}$ than for the other terms. The fit parameters for the individual contributions are summarized in Tables S12 and S13.

**Table S12.** Fit parameters of the EDA vs FIA values for the adsorption to SiO_2_.

| SiO_2_ | a | b |
| --- | --- | --- |
| E_bond_ | 0.419 | -66.139 |
| E_bond_ (without **1**-iPr, **1**-iBu) | 0.380 | -44.919 |
| ΔE_int_ | 0.671 | -92.602 |
| ΔE_int_ (without **1**-iPr, **1**-iBu) | 0.643 | -77.788 |
| ΔE_Pauli_ | 0.810 | -5.298 |
| ΔE_Pauli_ (without **1**-iPr, **1**-iBu) | 0.751 | 26.261 |
| ΔE_elstat_ | 0.921 | -106.966 |
| ΔE_elstat_ (without **1**-iPr, **1**-iBu) | 0.846 | -66.620 |
| ΔE_orb_ | 0.748 | -131.561 |
| ΔE_orb_ (without **1**-iPr, **1**-iBu) | 0.704 | -107.667 |

**Table S13.** Fit parameters of the EDA vs FIA values for the adsorption to the methoxy group of TMPS.

| TMPS | a | b |
| --- | --- | --- |
| E_bond_ | 0.376 | -49.428 |
| ΔE_int_ | 0.527 | -61.253 |
| ΔE_Pauli_ | 0.596 | 51.515 |
| ΔE_elstat_ | 0.672 | -32.525 |
| ΔE_orb_ | 0.598 | -96.941 |

The overall analysis of the individual EDA terms shows that $\Delta E_{\mathrm{orb}}$ exhibits the strongest correlation with the FIA, whereas $\Delta E_{\mathrm{Pauli}}$ shows the weakest correlation, both on SiO_2_ and the methoxy group of TMPS. In general, correlations are better on SiO_2_ than on TMPS. The poor correlation of $\Delta E_{\mathrm{Pauli}}$ is unsurprising, as it is dominated by steric effects of the precursors, which are not reflected in the FIA. Conversely, the stronger correlation of $\Delta E_{\mathrm{orb}}$ compared with $\Delta E_{\mathrm{elstat}}$ is expected because $\Delta E_{\mathrm{orb}}$ primarily reflects the Al–O bond, whereas $\Delta E_{\mathrm{elstat}}$ also includes additional electrostatic interactions of the precursor not captured by the FIA. These effects are particularly pronounced for the bulky precursor **3**.

8. **EDA of dimers**

Table S14 shows the EDAs of monomers and dimers of the chlorinated precursors **2** adsorbing to SiO_2_, Table S15 to the methoxy group of TMPS. The comparison of the bond distances of the dissociated Al-Cl dimer bond for both bonding situations is given in Table S16.

**Table S14.** EDA terms of dimers compared to monomers on SiO_2_.^[a]^

|  | **2**-Me_2_Cl | **2**-MeCl_2_ | **2**-Cl | **(2**-Me_2_Cl)_2_ | **(2**-MeCl_2_)_2_ | **(2**-Cl)_2_ |
| --- | --- | --- | --- | --- | --- | --- |
| ΔE_int_ | -177 | -213 | -239 | -126 | -175 | -208 |
| ΔE_int_ (disp) | -51 (29%) | -49 (23%) | -49 (21%) | -62 (49%) | -61 (35%) | -62 (30%) |
| ΔE_int_ (elec) | -126 (71%) | -164 (77%) | -190 (79%) | -64 (51%) | -114 (65%) | -146 (70%) |
| ΔE_Pauli_ | 324 | 373 | 391 | 283 | 355 | 397 |
| ΔE_elstat_ | -276 (61%) | -323 (60%) | -344 (59%) | -214 (62%) | -281 (60%) | -323 (59%) |
| ΔE_orb_ | -174 (39%) | -214 (40%) | -237 (41%) | -133 (38%) | -188 (40%) | -220 (41%) |
| ΔE_prep_ (mol) | 28 | 35 | 42 | 49 | 64 | 74 |
| ΔE_prep_ (surf) | 47 | 49 | 55 | 30 | 41 | 48 |
| ΔE_prep_ (total) | 75 | 84 | 97 | 79 | 105 | 122 |
| E_bond_ (PAW)^[b]^ | -103 (-106) | -128 (-134) | -142 (-148) | -48 (-50) | -70 (-74) | -86 (-91) |
| R (O-Al) | 1.982 | 1.930 | 1.891 | 2.095 | 2.015 | 1.956 |

[a] Energies in kJ∙mol^-1^, bond lengths in Å

[b] Energies calculated in VASP with a plane wave basis are shown in parentheses. Values for **2**-Cl and (**2**-Cl)_2_ are already published in ref.^1^

**Table S15.** EDA terms of dimers compared to monomers on TMPS on SiO_2_.^[a]^

|  | **2**-Me_2_Cl | **2**-MeCl_2_ | **2**-Cl | **(2**-Me_2_Cl)_2_ | **(2**-MeCl_2_)_2_ | **(2**-Cl)_2_ |
| --- | --- | --- | --- | --- | --- | --- |
| ΔE_int_ | -143 | -173 | -204 | -126 | -175 | -226 |
| ΔE_int_ (disp) | -48 (34%) | -47 (27%) | -48 (24%) | -56 (44%) | -55 (31%) | -54 (24%) |
| ΔE_int_ (elec) | -95 (66%) | -126 (73%) | -156 (76%) | -70 (56%) | -120 (69%) | -172 (76%) |
| ΔE_Pauli_ | 288 | 330 | 353 | 254 | 328 | 365 |
| ΔE_elstat_ | -238 (62%) | -280 (61%) | -306 (60%) | -202 (62%) | -270 (60%) | -317 (59%) |
| ΔE_orb_ | -145 (38%) | -176 (39%) | -203 (40%) | -122 (38%) | -178 (40%) | -220 (41%) |
| ΔE_prep_ (mol) | 29 | 33 | 39 | 66 | 98 | 121 |
| ΔE_prep_ (surf) | 16 | 22 | 25 | 13 | 21 | 29 |
| ΔE_prep_ (total) | 45 | 55 | 64 | 79 | 119 | 150 |
| E_bond_ (PAW)^[b]^ | -98 (-95) | -118 (-116) | -140 (-138) | -47 (-43) | -56 (-53) | -76 (-74) |
| R (O-Al) | 2.015 | 1.966 | 1.927 | 2.117 | 1.985 | 1.918 |

[a] Energies in kJ∙mol^-1^, bond lengths in Å

[b] Energies calculated in VASP with a plane wave basis are shown in parentheses

**Table S16.** Bond length comparison of the broken Al-Cl dimer bond for the adsorption of the dimers to SiO_2_ and to the methoxy group of TMPS.^[a]^

|  | d(Al-Cl) SiO_2_ | d(Al-Cl) TMPS |
| --- | --- | --- |
| **(2**-Me_2_Cl)_2_ | 2.621 | 2.826 |
| **(2**-MeCl_2_)_2_ | 2.628 | 3.140 |
| **(2**-Cl)_2_ | 2.554 | 3.358 |

[a] Bond lengths in Å

The bond length comparison shows that the dimer opening is more complete when the precursor adsorbs to the methoxy group than on the surface.

9. **EDA of precursors adsorbing in between SMI-layer**

**Figure S13.** Adsorption structures and energies of the monomers that can adsorb between the TMPS layer. Dispersion contribution in brackets. Figure reproduced from Maue et al.,^7^ 2026, licensed under CC BY 4.0.

**Figure S14.** Adsorption structures and energies of the chlorinated dimers that can adsorb between the TMPS layer. Dispersion contribution in brackets. Figure reproduced from Maue et al.,^7^ 2026, licensed under CC BY 4.0.

In addition to adsorption on the methoxy group of a single TMPS molecule, we also examined adsorption within the TMPS layer on a partially covered surface. Table S17 presents the EDA terms for the precursors, including dimers capable of adsorbing between the inhibitors.

**Table S17.** EDA of monomers and dimers adsorbing between TMPS.^[a]^

|  | **1**-Me | **2**-Me_2_Cl | **2**-MeCl_2_ | **2**-Cl | (**2**-Me_2_Cl)_2_ | (**2**-MeCl_2_)_2_ | (**2**-Cl)_2_ |
| --- | --- | --- | --- | --- | --- | --- | --- |
| ΔE_int_ | -161 | -189 | -295 | -395 | -247 | -314 | -535 |
| ΔE_int_ (disp) | -107 (66%) | -106 (56%) | -104 (35%) | -102 (26%) | -137 (55%) | -133 (42%) | -131 (24%) |
| ΔE_int_ (elec) | -54 (34%) | -83 (44%) | -191 (65%) | -293 (74%) | -110 (45%) | -181 (58%) | -404 (76%) |
| ΔE_Pauli_ | 476 | 513 | 612 | 748 | 574 | 607 | 884 |
| ΔE_elstat_ | -328 (62%) | -367 (62%) | -471 (59%) | -589 (57%) | -415 (61%) | -468 (59%) | -701 (54%) |
| ΔE_orb_ | -202 (38%) | -229 (38%) | -332 (41%) | -452 (43%) | -269 (39%) | -320 (41%) | -587 (46%) |
| ΔE_prep_ (mol) | 64 | 70 | 105 | 162 | 158 | 175 | 294 |
| ΔE_prep_ (surf) | 139 | 146 | 178 | 199 | 155 | 176 | 199 |
| ΔE_prep_ (total) | 203 | 216 | 283 | 361 | 313 | 351 | 493 |
| E_bond_ (PAW)^[b]^ | 43 (46) | 27 (29) | -12 (-12) | -34 (-34) | 66 (72) | 37 (44) | -42 (-37) |
| R (O-Al) | 2.060 | 2.012 | 1.950 | 1.939 | 1.968 | 1.920 | 1.940 |

[a] Energies in kJ∙mol^-1^, bond lengths in Å

[b] Energies calculated in VASP with a plane wave basis are shown in parentheses. These values are already published in ref.^7^

In the following, we discuss the trends in the EDA contributions with increasing numbers of chlorine substituents and the effects of dimerization for precursors adsorbed between the SMIs. Figure S15a presents the EDA terms of the chlorinated precursors relative to **1**-Me, while Figure S15b shows the energy contributions of the dimers relative to their corresponding monomers.

**Figure S15.** (a) pEDA terms of monomers of group **2** relative to **1**-Me adsorbing to the surface between TMPS. (b) pEDA terms of the dimers of group **2** relative to their corresponding monomers with the difference in bond distance between dimers and monomers Δd.

For the monomers (Figure S15a), ${\Delta E}_{\mathrm{int}}\left( \mathrm{disp} \right)$ remains relatively constant with increasing number of chlorine atoms, differing by only 5 kJ·mol^-1^ between **1**-Me and **2**-Cl. In contrast, $\Delta E_{\mathrm{Pauli}}$ steadily increases with chlorination, reaching 748 kJ·mol^-1^ for **2**-Cl, which is 272 kJ·mol^-1^ higher than for **1**-Me. The $\Delta E_{\mathrm{elstat}}$ and $\Delta E_{\mathrm{orb}}$ contributions also increase continuously, by 261 kJ·mol^-1^ and 250 kJ·mol^-1^, respectively, from **1**-Me to **2**-Cl. $\Delta E_{\mathrm{prep}}$ of both the precursor and the surface rise significantly, by 98 kJ·mol^-1^ for the precursor and 60 kJ·mol^-1^ for the surface. As observed for adsorption on the pristine SiO_2_ surface, both the stabilizing and destabilizing contributions increase with the number of chlorine atoms. Overall, $E_{\mathrm{bond}}$ is stabilized, changing from 43 kJ·mol^-1^ for **1**-Me to –34 kJ·mol^-1^ for **2**-Cl, accompanied by a bond length decrease of 0.121 Å. This shows that increasing chlorination enhances adsorption, reaching exothermic adsorption for **2**-MeCl_2_, whereas **1**-Me and **2**-Me_2_Cl require energy to adsorb between the inhibitors. In summary, the trends with respect to the number of chlorine atoms closely mirror those observed on the free surface.

Dimerization (Figure S15b) leads to a destabilization of $E_{\mathrm{bond}}$ relative to the monomer for (**2**-Me_2_Cl)_2_ and (**2**-MeCl_2_)_2_, although this effect is less pronounced than on pristine SiO_2_. In contrast, (**2**-Cl)_2_ exhibits a stabilized $E_{\mathrm{bond}}$ compared to its corresponding monomer. Unlike the behavior on the free SiO_2_ surface, $\Delta E_{\mathrm{int}}$ is enhanced upon dimerization, and the weakened $E_{\mathrm{bond}}$ primarily results from an increased $\Delta E_{\mathrm{prep}}$. For (**2**-Me_2_Cl)_2_ and (**2**-Cl)_2_, both $\Delta E_{\mathrm{elstat}}$ and $\Delta E_{\mathrm{orb}}$ contributions are significantly larger than for the monomers, with $\Delta E_{\mathrm{elstat}}$ increasing by 48 and 112 kJ·mol^-1^, respectively. The dimer–surface bond lengths differ by less than 0.05 Å from those of the monomers, indicating similar bonding geometries. This behavior can be attributed to stronger structural deformation upon adsorption between the inhibitors, leading to complete dimer opening—unlike on the pristine surface—and consequently to stronger Al–O interactions, as supported by the elongated Al–Cl bond lengths listed in Table S18. Although the attractive interactions between the dimers and the surface (including inhibitors) are stronger than for the monomers under these conditions, the substantial energy required for dimer opening and deformation outweighs this stabilization, resulting in overall weaker adsorption of the dimers.

**Table S18.** Bond length comparison of the broken Al-Cl dimer bond for the adsorption of the dimers on free SiO_2_ and on SiO_2_ between the inhibitors.^[a]^

|  | d(Al-Cl) free | d(Al-Cl) covered |
| --- | --- | --- |
| (**2**-Me_2_Cl)_2_ | 2.621 | 3.956 |
| (**2**-MeCl_2_)_2_ | 2.628 | 4.133 |
| (**2**-Cl)_2_ | 2.554 | 4.520 |

[a] Bond lengths in Å

**References**

(1)	Maue, P.; Chantraine, É.; Pieck, F.; Tonner-Zech, R. Accurately Computed Dimerization Trends of ALD Precursors and Their Impact on Surface Reactivity in Area-Selective Atomic Layer Deposition. *Chem. Mater.* **2025**, *37* (3), 975–988. https://doi.org/10.1021/acs.chemmater.4c02557.

(2)	Sun, X.; Soini, T. M.; Poater, J.; Hamlin, T. A.; Bickelhaupt, F. M. PyFrag 2019—Automating the Exploration and Analysis of Reaction Mechanisms. *J. Comput. Chem.* **2019**, *40* (25), 2227–2233. https://doi.org/10.1002/jcc.25871.

(3)	SCM. Amsterdam Modeling Suite. http://www.scm.com (accessed 2024-07-03).

(4)	Perdew, J. P.; Ernzerhof, M.; Burke, K. Rationale for Mixing Exact Exchange with Density Functional Approximations. *J. Chem. Phys.* **1996**, *105* (22), 9982–9985. https://doi.org/10.1063/1.472933.

(5)	Van Lenthe, E.; Baerends, E. J. Optimized Slater‐type Basis Sets for the Elements 1–118. *J. Comput. Chem.* **2003**, *24* (9), 1142–1156. https://doi.org/10.1002/jcc.10255.

(6)	Chong, D. P.; Van Lenthe, E.; Van Gisbergen, S.; Baerends, E. J. Even‐tempered Slater‐type Orbitals Revisited: From Hydrogen to Krypton. *J. Comput. Chem.* **2004**, *25* (8), 1030–1036. https://doi.org/10.1002/jcc.20030.

(7)	Maue, P.; Pieck, F.; Tonner-Zech, R. Role of Idealized Surface Representations in Computational Area-Selective Atomic Layer Deposition: Assessing Trimethoxypropylsilane Blocking Performance for Al_2_O_3_ Deposition with Crystalline SiO_2_. *J. Vac. Sci. Technol. A* **2026**, *44* (2), 022404. https://doi.org/10.1116/6.0004954.
